# Design, Synthesis, and Structure–Activity Relationships of Thiazole Analogs as Anticholinesterase Agents for Alzheimer’s Disease

**DOI:** 10.3390/molecules25184312

**Published:** 2020-09-20

**Authors:** Begüm Nurpelin Sağlık, Derya Osmaniye, Ulviye Acar Çevik, Serkan Levent, Betül Kaya Çavuşoğlu, Yusuf Özkay, Zafer Asım Kaplancıklı

**Affiliations:** 1 Department of Pharmaceutical Chemistry, Faculty of Pharmacy, Anadolu University, 26470 Eskişehir, Turkey; bnsaglik@anadolu.edu.tr (B.N.S.); dosmaniye@anadolu.edu.tr (D.O.); uacar@anadolu.edu.tr (U.A.Ç.); serkanlevent@anadolu.edu.tr (S.L.); yozkay@anadolu.edu.tr (Y.Ö.); 2 Doping and Narcotic Compounds Analysis Laboratory, Faculty of Pharmacy, Anadolu University, 26470 Eskişehir, Turkey; 3 Department of Pharmaceutical Chemistry, Faculty of Pharmacy, Zonguldak Bülent Ecevit University, 67600 Zonguldak, Turkey; betul.kcavusoglu@beun.edu.tr

**Keywords:** ADME parameters, Alzheimer’s disease, anticholinesterase enzyme activity, molecular docking, thiazole

## Abstract

Dementia is a neurological condition commonly correlated with Alzheimer’s disease (AD), and it is seen with many other central nervous system (CNS) disorders. The restricted number of medications is not appropriate to offer enough relief to enhance the quality of life of patients suffering from this symptom; thus, all therapeutic choices should be carefully assessed. In this study, new thiazolylhydrazone derivatives (**2a**–**2l**) were designed and synthesized based on the cholinergic hypothesis. Their chemical structures were confirmed by ^1^H NMR, ^13^C NMR, and HRMS spectrometric techniques. The ADME (absorption, distribution, metabolism, elimination) parameters of the synthesized compounds were predicted by using QikProp 4.8 software. It was concluded that all compounds presented satisfactory drug-like characteristics. Furthermore, their inhibitory activities against acetylcholinesterase (AChE) and butyrylcholinesterase (BChE) in vitro were also tested by modified the Ellman spectrophotometric method. According to the results, all compounds showed a weak inhibitory effect on BChE. On the other hand, most of the compounds (**2a**, **2b**, **2d**, **2e**, **2g**, **2i**, and **2j**) had a certain AChE inhibitory activity, and the IC_50_ values of them were calculated as 0.063 ± 0.003, 0.056 ± 0.002, 0.147 ± 0.006, 0.040 ± 0.001, 0.031 ± 0.001, 0.028 ± 0.001, and 0.138 ± 0.005 µM, respectively. Among these derivatives, compound **2i** was found to be the most active agent in the series with an IC_50_ value of 0.028 ± 0.001 µM, which indicated an inhibition profile at a similar rate as the reference drug, donepezil. The potential binding modes of compounds **2a**, **2b**, **2e**, **2g**, and **2i** with AChE were investigated and compared with each other by the molecular docking studies. The results showed that these compounds were strongly bound up with the AChE enzyme active site with the optimal conformations.

## 1. Introduction

Alzheimer’s disease (AD), the most common type of dementia [1], is an irreversible, progressive brain disease that has destructive effects on memory and thinking [2]. The most frequent symptoms of AD are impairment in the formation of the short-term memory, problems in language skills, and attention deficits. Personality change, deterioration of visuospatial skills, anxiety, and depression are common during the course of the disease. Patients eventually lose the ability to be self-supportive and become bedridden. The survival of the disease is 8–10 years, and death usually occurs due to complications that result from immobility and malnutrition [3].

Although there are many hypotheses about the pathophysiology of the disease, its pathogenesis cannot be fully explained [4]. The cholinergic system [5], beta amyloid (Aβ) protein [6], tau protein, oxidative stress [7], inflammation [8], and toxic metal ions [9] are the main hypotheses that explain the causes and progression of AD. In line with these hypotheses, increasing cholinergic activity with cholinesterase inhibitory compounds [10], inhibiting Aβ aggregate formation [11], and reducing the hyperphosphorylation of tau protein [12] are the main treatment approaches for the disease. Studies for the treatment of AD have focused primarily on cholinesterase (ChE) inhibitors, which ensure the regulation of cholinergic conduction. The acetylcholine (ACh) is the main neurotransmitter of the cholinergic system, and its hydrolysis into choline and acetic acid is catalyzed by acetylcholinesterase (AChE). The cholinergic hypothesis is based on the presumption that the inhibition of AChE would prevent the hydrolysis of ACh, so that the level of ACh in cholinergic synapses is increased. Donepezil, galantimine, and rivastigmine [13] are cholinesterase inhibitors that have been used as drugs approved by the Food and Drug Administration (FDA) for AD [14]. However, the drugs used in the treatment of AD are only for relieving the symptoms of the disease, and a drug that stops the progression of the disease has not yet been developed. In addition, the currently used drugs have side effects that limit their clinical use. For this reason, scientific studies for the discovery of stronger drugs with fewer side effects for the treatment of AD remain important and continue intensely [15,16,17,18].

It is well-known that the AChE enzyme active site structure is important to the development of new ChE inhibitors. The catalytic anionic site (CAS) and the peripheral anionic binding site (PAS) form the structure of the AChE active site. Chemically, the aromatic groups of the compounds bind to the PAS region, whereas the moieties carrying a basic center interact with the CAS [19,20]. Dual binding inhibitors such as donepezil bind to both sites; therefore, these types of compounds can show a very potent AChE inhibition profile [21,22,23,24].

**Figure 1:**
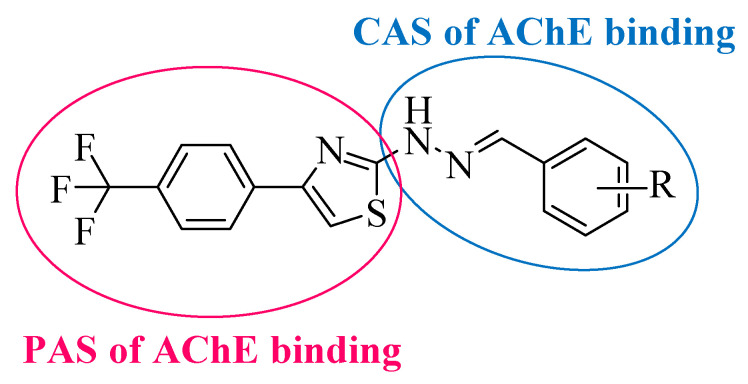
The chemical parts of the target compounds **2a**–**2l** according to the binding to the catalytic anionic site (CAS) and peripheral anionic binding site (PAS) regions.

**Scheme 1:**
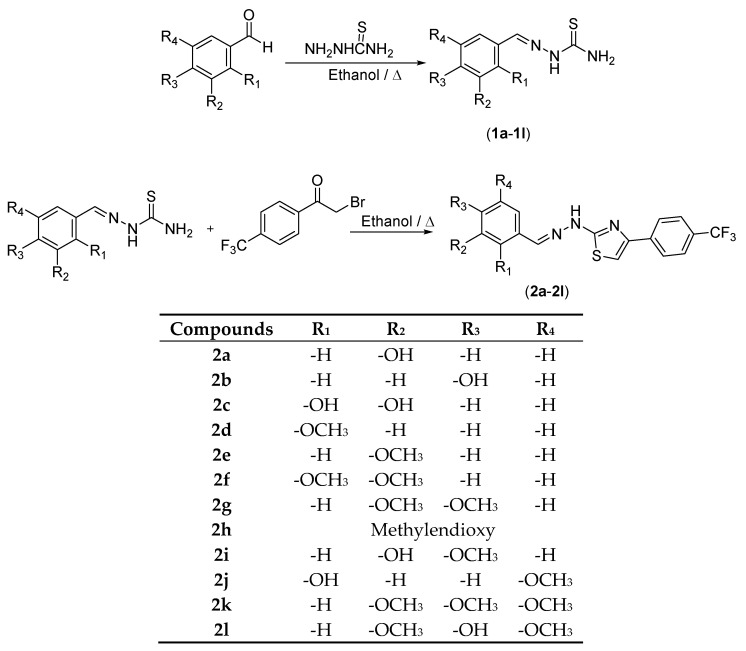
Synthesis way of the compounds **2a**–**2l**.

Heterocyclic rings are frequently used as the main structure in the design of biological compounds [25]. It has been demonstrated in several studies that compounds carrying a thiazole ring are effective against neurodegenerative disorders caused by low cholinergic neurotransmitter levels [26,27,28,29,30,31]. In this paper, we aim to present the new thiazole derivatives as ChE inhibitors. In accordance with this purpose, the thiazole ring and hydrazine moiety, which is thought to act as a basic center, are gathered structurally. So, novel 3-((2-(4-(4-(trifluoromethyl)phenyl)thiazol-2-yl)hydrazineylidene)methyl)-substituedphenyl derivatives were designed and synthesized. It is believed that the lipophilic 4-(4-(trifluoromethyl)phenyl)thiazole group can bind to the PAS region; however, the binding to the CAS of the enzyme active site can be provided by the benzylidenehydrazine moiety (Figure 1). Moreover, the hydroxyl and methoxy groups were selected as functional groups inspired by the methoxy groups in donepezil molecule (Scheme 1). Thus, it was aimed to discuss the contribution of functional groups such as these.

## 2. Materials and Methods

### 2.1. Chemistry

All the chemicals used in the synthesis were obtained from Merck (Darmstadt, Germany) or Sigma-Aldrich (St. Louis, MO, USA). A MP90 digital melting point apparatus (Mettler Toledo, OH, USA) was used to determine the melting points of the resulting compounds and was presented uncorrected. The R_f_ values of the synthesized compounds were measured using the solution system of petroleum ether:ethyl acetate (3:1). The obtained view of thin-layer chromatography was also added in the Supplementary Material Figure S7. ^1^H NMR and ^13^C NMR spectra were recorded by a Bruker 300 MHz and 75 MHz digital FT-NMR spectrometer (Bruker Bioscience, Billerica, MA, USA) in DMSO-*d*_6_, respectively. In the NMR spectra, splitting patterns were determined and recognized as follows: s: singlet, d: doublet, t: triplet, dd: double doublet, and m: multiplet. Coupling constants (*J*) were reported in units of Hertz (Hz). Mass spectra were recorded on an LCMS-IT-TOF (Shimadzu, Kyoto, Japan) equipped with a PDA detector (Supplementary Material Figure S8). Silica gel 60 F254 with thin-layer chromatography (Merck KGaA, Darmstadt, Germany) was used to check the purity of compounds.

#### 2.1.1. General Procedure for the Synthesis of the Compounds

##### Synthesis of Substituted Thiosemicarbazones (**1a**–**1l**)

A mixture of appropriately substituted benzaldehyde (0.016 mol) and thiosemicarbazide (1.456 g, 0.016 mol) was refluxed in EtOH (100 mL) for 3 h. After completion of the reaction, the mixture was cooled in an ice bath, and the precipitated product was filtered, dried, and recrystallized from the EtOH.

##### General Procedures of Target Compounds (**2a**–**2l**)

Corresponding substituted thiosemicarbazone (**1a**–**1l**) (0.001 mol) and 2-bromo-1-(4-(trifluoromethyl)phenyl)ethan-1-one (0.001 mol) in EtOH (40 mL) were refluxed for 4 h. The mixture was cooled in an ice bath, and the precipitated product was filtered, dried, and recrystallized from the EtOH.

*3-[(2-{4-[4-(Trifluoromethyl)phenyl]thiazol-2-yl}hydrazineylidene)methyl]phenol* (**2a**) Yield: 81%. R_f_ = 0.604. M.P.: 249.2–251.8 °C. Purity: 96.8%. ^1^H-NMR (300 MHz, DMSO-*d*_6_): 6.78–6.82 (1H, m, monosubstituted benzene), 7.06 (1H, d, *J* = 7.7 Hz, monosubstituted benzene), 7.12–7.13 (1H, m, monosubstituted benzene), 7.24 (1H, t, *J* = 7.8 Hz, monosubstituted benzene), 7.59 (1H, s), 7.78 (2H, d, *J* = 8.3 Hz, 1,4-disubstituted benzene), 7.98 (1H, s), 8.08 (2H, d, *J* = 8.0 Hz, 1,4-disubstituted benzene), 9.49 (1H, br.s., -OH), 12.23 (1H, br.s., -NH). ^13^C-NMR (75 MHz, DMSO-*d*_6_): δ = 106.9, 112.4, 117.2, 118.4, 119.4, 123.0, 124.8 (*J* = 269.9 Hz), 126.0 (*J* = 3.6 Hz), 126.5, 128.2 (*J* = 31.5 Hz), 130.3, 135.9, 138.7, 142.3, 149.2, 158.1, 168.9. HRMS *(m*/*z*): [M+H]^+^ calcd for C_17_H_12_N_3_OF_3_S: 364.0726; found: 364.0727.

*4-[(2-{4-[4-(Trifluoromethyl)phenyl]thiazol-2-yl}hydrazineylidene)methyl]phenol* (**2b**) Yield: 79%. R_f_ = 0.472. M.P.: 244.1–247.0 °C. Purity: 90.6%. ^1^H-NMR (300 MHz, DMSO-*d*_6_): 6.85 (2H, d, *J* = 8.6 Hz, 1,4-disubstituted benzene), 7.52 (2H, t, *J* = 8.7 Hz, 1,4-disubstituted benzene), 7.56 (1H, s), 7.78 (2H, d, *J* = 8.3 Hz, 1,4-disubstituted benzene), 7.99 (1H, s), 8.07 (2H, d, *J* = 8.1 Hz, 1,4-disubstituted benzene), 9.28 (1H, br.s., -OH), 12.04 (1H, br.s., -NH). ^13^C-NMR (75 MHz, DMSO-*d*_6_): δ = 106.6, 116.2, 124.8 (*J* = 270.5 Hz), 125.6, 126.0 (*J* = 3.7 Hz), 126.5, 128.4 (*J* = 31.3 Hz), 128.7, 138.5, 142.9, 148.8, 159.3, 169.1. HRMS (*m*/*z*): [M+H]^+^ calcd for C_17_H_12_N_3_OF_3_S: 364.0726; found: 364.0739.

*3-[(2-{4-[4-(Trifluoromethyl)phenyl]thiazol-2-yl}hydrazineylidene)methyl]benzene-1,2-diol* (**2c**) Yield: 75%. R_f_ = 0.417. M.P.: 234.2–236.8 °C. Purity: 98.5%. ^1^H-NMR (300 MHz, DMSO-*d*_6_): 6.72 (1H, t, *J* = 7.8 Hz, 1,2,3-trisubstituted benzene), 6.84 (1H, dd, *J*_1_ = 1.6 Hz, *J*_2_ = 7.8 Hz, 1,2,3-trisubstituted benzene), 7.12 (1H, dd, *J*_1_ = 1.6 Hz, *J*_2_ = 7.9 Hz, 1,2,3-trisubstituted benzene), 7.57 (1H, s), 7.78 (2H, d, *J* = 8.3 Hz, 1,4-disubstituted benzene), 8.08 (2H, d, *J* = 8.0 Hz, 1,4-disubstituted benzene), 8.36 (1H, s), 9.33 (1H, br.s., -OH), 12.25 (1H, br.s., -NH). ^13^C-NMR (75 MHz, DMSO-*d*_6_): δ = 106.5, 116.6, 117.3, 119.7, 120.9, 124.8 (*J* = 269.9 Hz), 126.0 (*J* = 3.6 Hz), 126.5, 128.0 (*J* = 31.4 Hz), 138.7, 141.1, 145.1, 146.0, 149.4, 168.6. HRMS (*m*/*z*): [M+H]^+^ calcd for C_17_H_12_N_3_O_2_F_3_S: 380.0675; found: 380.0675.

*2-[2-(2-Methoxybenzylidene)hydrazineyl]-4-[4-(trifluoromethyl)phenyl]thiazole* (**2d**) Yield: 78%. R_f_ = 0.833. M.P.: 223.0–225.9 °C. Purity: 92.4%. ^1^H-NMR (300 MHz, DMSO-*d*_6_): 3.84 (3H, s, -OCH_3_), 7.00 (1H, t, *J* = 7.6 Hz, 1,2-disubstituted benzene), 7.08 (1H, d, *J* = 8.1 Hz, 1,2-disubstituted benzene), 7.33–7.39 (1H, m, 1,2-disubstituted benzene), 7.56 (1H, s), 7.75 (2H, d, *J* = 8.5 Hz, 1,4-disubstituted benzene), 7.79–7.83 (1H, m, 1,2-disubstituted benzene), 8.04 (2H, d, *J* = 8.0 Hz, 1,4-disubstituted benzene), 8.38 (1H, s), 12.29 (1H, br.s., -NH). ^13^C-NMR (75 MHz, DMSO-*d*_6_): δ = 56.1, 106.9, 112.2, 121.2, 122.6, 124.7 (*J* = 265.2 Hz), 125.3, 126.0 (*J* = 3.6 Hz), 128.0 (*J* = 31.4 Hz), 128.9, 131.3, 137.7, 138.6, 149.1, 157.6, 168.9. HRMS (*m*/*z*): [M+H]^+^ calcd for C_18_H_14_N_3_OF_3_S: 378.0882; found: 378.0887.

*2-[2-(3-Methoxybenzylidene)hydrazineyl]-4-[4-(trifluoromethyl)phenyl]thiazole* (**2e**) Yield: 80%. R_f_ = 0.799. M.P.: 238.8–241.0 °C. Purity: 94.5%. ^1^H-NMR (300 MHz, DMSO-*d*_6_): 3.80 (3H, s, -OCH_3_), 6.94–6.98 (1H, m, 1,3-disubstituted benzene), 7.20–7.25 (2H, m, 1,4-disubstituted benzene), 7.35 (1H, t, *J* = 7.8 Hz, 1,3-disubstituted benzene), 7.59 (1H, s), 7.76 (1H, d, *J* = 8.3 Hz, 1,3-disubstituted benzene), 7.84 (1H, d, *J* = 8.4 Hz, 1,3-disubstituted benzene), 8.02 (1H, s), 8.06 (2H, d, *J* = 7.9 Hz, 1,4-disubstituted benzene), 12.35 (1H, br.s., -NH). ^13^C-NMR (75 MHz, DMSO-*d*_6_): δ = 55.5, 107.0, 111.6, 115.6, 119.3, 124.8 (*J* = 268.6 Hz), 126.0 (*J* = 3.6 Hz), 127.9 (*J* = 31.4 Hz), 128.9, 130.4, 136.1, 138.7, 141.8, 149.3, 159.9, 168.9. HRMS (*m*/*z*): [M+H]^+^ calcd for C_18_H_14_N_3_OF_3_S: 378.0882; found: 378.0887.

*2-[2-(2,3-Dimethoxybenzylidene)hydrazineyl]-4-[4-(trifluoromethyl)phenyl]thiazole* (**2f**) Yield: 83%. R_f_ = 0.826. M.P.: 212.5–214.9 °C. Purity: 96.7%. ^1^H-NMR (300 MHz, DMSO-*d*_6_): 3.76 (3H, s, -OCH_3_), 3.81 (3H, s, -OCH_3_), 7.03–7.08 (1H, m, 1,2,3-trisubstituted benzene), 7.09 (1H, d, *J* = 7.7 Hz, 1,2,3-trisubstituted benzene), 7.34–7.40 (1H, m, 1,2,3-trisubstituted benzene), 7.55 (1H, s), 7.74 (1H, d, *J* = 8.4 Hz, 1,4-disubstituted benzene), 7.80 (1H, d, *J* = 8.4 Hz, 1,4-disubstituted benzene), 8.03–8.07 (2H, m, 1,2,3-trisubstituted benzene, Ar-H), 8.32 (2H, d, *J* = 6.2 Hz, 1,2,3-trisubstituted benzene). ^13^C-NMR (75 MHz, DMSO-*d*_6_): δ = 56.1, 61.5, 93.4, 106.9, 114.1, 116.9, 124.5 (*J* = 265.4 Hz), 124.8, 126.5 (*J* = 3.6 Hz), 128.0 (*J* = 30.5 Hz), 128.8, 137.7, 138.6, 147.7, 149.1, 153.1, 167.6. HRMS (*m*/*z*): [M+H]^+^ calcd for C_19_H_16_N_3_O_2_F_3_S: 408.0988; found: 408.0989.

*2-[2-(3,4-Dimethoxybenzylidene)hydrazineyl]-4-[4-(trifluoromethyl)phenyl]thiazole* (**2g**) Yield: 77%. R_f_ = 0.479. M.P.: 198.6–200.3 °C. Purity: 90.3%. ^1^H-NMR (300 MHz, DMSO-*d*_6_): 3.78 (3H, s, -OCH_3_), 3.81 (3H, s, -OCH_3_), 7.00 (1H, d, *J* = 8.4 Hz, 1,3,4-trisubstituted benzene), 7.15–7.19 (1H, m, 1,3,4-trisubstituted benzene), 7.25–7.27 (1H, m, 1,3,4-trisubstituted benzene), 7.55 (1H, s), 7.75 (2H, d, *J* = 8.4 Hz, 1,4-disubstituted benzene), 7.99 (1H, s), 8.05 (2H, d, *J* = 8.0 Hz, 1,4-disubstituted benzene), 12.31 (1H, br.s., -NH). ^13^C-NMR (75 MHz, DMSO-*d*_6_): δ = 55.7, 55.9, 93.0, 106.7, 108.7, 112.0, 120.8, 124.7 (*J* = 263.6 Hz), 126.0 (*J* = 3.5 Hz), 127.9 (*J* = 31.6 Hz), 128.8, 138.6, 142.4, 146.6, 149.4, 150.6, 169.0. HRMS (*m*/*z*): [M+H]^+^ calcd for C_19_H_16_N_3_O_2_F_3_S: 408.0988; found: 408.0989.

*2-[2-(Benzo[1,3]dioxol-5-ylmethylene)hydrazineyl]-4-[4-(trifluoromethyl)phenyl]thiazole* (**2h**) Yield: 82%. R_f_ = 0.813. M.P.: 237.2–239.9 °C. Purity: 90.2%. ^1^H-NMR (300 MHz, DMSO-*d*_6_): 6.08 (2H, s, benzo[1,3]dioxol), 6.10 (1H, d, *J* = 8.0 Hz, benzo[1,3]dioxol), 7.10–7.13 (1H, m, benzo[1,3]dioxol), 7.23 (1H, d, *J* = 1.3 Hz, benzo[1,3]dioxol), 7.57 (1H, s), 7.76 (2H, d, *J* = 8.4 Hz, 1,4-disubstituted benzene), 7.96 (1H, s), 8.05 (2H, d, *J* = 8.0 Hz, 1,4-disubstituted benzene), 12.20 (1H, br.s., -NH). ^13^C-NMR (75 MHz, DMSO-*d*_6_): δ = 101.9, 105.0, 106.8, 109.0, 122.6, 124.8 (*J* = 269.9 Hz), 126.0 (*J* = 3.6 Hz), 126.5, 127.9 (*J* = 31.6), 128.9, 138.7, 141.9, 148.4, 148.9, 149.2, 169.0. HRMS (*m*/*z*): [M+H]^+^ calcd for C_18_H_12_N_3_O_2_F_3_S: 392.0675; found: 392.0681.

*2-Methoxy-5-[(2-{4-[4-(trifluoromethyl)phenyl]thiazol-2-yl}hydrazineylidene)methyl]phenol* (**2i**) Yield: 78%. R_f_ = 0.375. M.P.: 226.5–229.8 °C. Purity: 91.1%. ^1^H-NMR (300 MHz, DMSO-*d*_6_): 3.79 (3H, s, -OCH_3_), 6.94 (1H, d, *J* = 8.4 Hz, 1,3,4-trisubstituted benzene), 6.98–7.01 (1H, m, 1,3,4-trisubstituted benzene), 7.19–7.20 (1H, m, 1,3,4-trisubstituted benzene), 7.54 (1H, s), 7.75 (1H, d, *J* = 8.4 Hz, 1,4-disubstituted benzene), 7.82 (1H, d, *J* = 8.4 Hz, 1,4-disubstituted benzene), 7.93 (1H, s), 8.05 (2H, d, *J* = 8.2 Hz, 1,4-disubstituted benzene). ^13^C-NMR (75 MHz, DMSO-*d*_6_): δ = 55.9, 92.9, 106.6, 112.1, 112.3, 119.9, 124.8 (*J* = 269.1 Hz), 126.0 (*J* = 3.6 Hz), 127.5, 128.5 (*J* = 31.6 Hz), 128.8, 138.6, 142.7, 147.2, 149.7, 169.0. HRMS (*m*/*z*): [M+H]^+^ calcd for C_18_H_14_N_3_O_2_F_3_S: 394.0832; found: 394.0834.

*4-Methoxy-2-[(2-{4-[4-(trifluoromethyl)phenyl]thiazol-2-yl}hydrazineylidene)methyl]phenol* (**2j**) Yield: 75%. R_f_ = 0.688. M.P.: 252.8–255.1 °C. Purity: 93.2%. ^1^H-NMR (300 MHz, DMSO-*d*_6_): 3.72 (3H, s, -OCH_3_), 6.83–6.84 (2H, m, 1,2,5-trisubstituted benzene), 7.18–7.19 (1H, m, 1,2,5-trisubstituted benzene), 7.57 (1H, s), 7.76 (1H, d, *J* = 8.4 Hz, 1,4-disubstituted benzene), 8.06 (2H, d, *J* = 8.1 Hz, 1,4-disubstituted benzene), 8.31 (1H, s), 9.67 (1H, br.s., -OH), 12.26 (1H, br.s., -NH). ^13^C-NMR (75 MHz, DMSO-*d*_6_): δ = 55.7, 106.7, 109.9, 117.5, 117.6, 120.8, 124.3 (*J* = 270.3 Hz), 126.0 (*J* = 3.6 Hz), 126.5, 128.5 (*J* = 31.6 Hz), 138.7, 139.7, 149.4, 150.5, 152.7, 168.7. HRMS (*m*/*z*): [M+H]^+^ calcd for C_18_H_14_N_3_O_2_F_3_S: 394.0832; found: 394.0844.

*4-[4-(Trifluoromethyl)phenyl]-2-[2-(3,4,5-trimethoxybenzylidene)hydrazineyl]thiazole* (**2k**) Yield: 79%. R_f_ = 0.493. M.P.: 141.9–143.8 °C. Purity: 91.7%. ^1^H-NMR (300 MHz, DMSO-*d*_6_): 3.69 (3H, s, -OCH_3_), 3.83 (6H, s, -OCH_3_), 6.98 (2H, s), 7.59 (1H, s), 7.76 (2H, d, *J* = 8.4 Hz, 1,4-disubstituted benzene), 7.98 (1H, s), 8.06 (2H, d, *J* = 8.1 Hz, 1,4-disubstituted benzene), 12.29 (1H, br.s., -NH). ^13^C-NMR (75 MHz, DMSO-*d*_6_): δ = 56.2, 56.3, 60.5, 103.9, 106.9, 124.3 (*J* = 270.2 Hz), 126.0 (*J* = 3.6 Hz), 126.4, 128.4 (*J* = 36.1 Hz), 130.3, 138.7, 139.0, 141.9, 149.3, 153.6, 168.9. HRMS (*m*/*z*): [M+H]^+^ calcd for C_20_H_18_N_3_O_3_F_3_S: 438.1094; found: 438.1089.

*2,6-Dimethoxy-4-[(2-{4-[4-(trifluoromethyl)phenyl]thiazol-2-yl}hydrazineylidene)methyl]phenol* (**2l**) Yield: 76%. R_f_ = 0.208. M.P.: 248.0–250.1 °C. Purity: 93.8%. ^1^H-NMR (300 MHz, DMSO-*d*_6_): 3.81 (6H, s, -OCH_3_), 6.95 (2H, s), 7.54 (1H, s), 7.76 (2H, d, *J* = 8.4 Hz, 1,4-disubstituted benzene), 7.94 (1H, s), 8.06 (2H, d, *J* = 8.1 Hz, 1,4-disubstituted benzene), 8.84 (1H, br.s., -OH), 12.13 (1H, br.s., -NH). ^13^C-NMR (75 MHz, DMSO-*d*_6_): δ = 56.3, 56.4, 104.2, 106.5, 124.3 (*J* = 270.3 Hz), 125.0, 126.0 (*J* = 3.6 Hz), 126.4, 128.4 (*J* = 31.6 Hz), 137.7, 138.8, 142.6, 148.6, 149.4, 169.0. HRMS (*m*/*z*): [M+H]^+^ calcd for C_19_H_16_N_3_O_3_F_3_S: 424.0937; found: 424.0943.

### 2.2. Cholinesterase Enzymes Inhibition Assay

The in vitro AChE and BChE enzymes inhibition potencies of the synthesized compounds (**2a**–**2l**) were evaluated according to the modified Ellman’s spectrophotometric method [32]. The reagents and materials used in the enzyme inhibition assay were supplied commercially from Sigma-Aldrich (St. Louis, MO, USA) and Fluka (Steinheim, Germany). The cholinesterase enzyme inhibition procedure was applied as previously reported in our research papers [33,34,35,36,37,38,39,40].

The percentage of inhibition results were displayed as the mean ± standard deviation (SD). Moreover, the IC_50_ values were calculated with the help of GraphPad ‘PRISM’ software (version 5.0) by using a dose–response curve achieved by plotting the percentage inhibition versus the log concentration.

### 2.3. Kinetic Studies of Enzyme Inhibition

The compound **2i**, which was found to be the most effective derivative in the series, was included in the enzyme kinetics study to assign the type of inhibition. For this purpose, this compound was prepared at different concentrations (IC_50_, 2xIC_50_ and IC_50_/2). Moreover, a substrate (ATC) was used at various concentrations (600, 300, 150, 75, 37.5, and 18.75 µM). The enzyme kinetics assay was carried out as in our previous publications [33,34,35,36,37,38,39,40]. Lineweaver–Burk plots were formed using Microsoft Office Excel 2013. The K_i_ values of the compound were easily calculated from the second plot with a common intercept on the *x*-axis (corresponding to −K_i_).

### 2.4. Prediction of ADME Parameters and BBB Permeability

In order to predict the pharmacokinetic profiles of synthesized compounds **2a**–**2l**, *QikProp 4.8* software [41] was used, and their physicochemical parameters were calculated via the in silico method.

### 2.5. In Vitro BBB Permeability Assay

In order to observe the BBB crossing ability of the most active compound **2i**, the parallel artificial membrane permeability assay (PAMPA) was performed as previously described [42].

### 2.6. Molecular Docking

A structure-based in silico procedure was applied to discover the binding modes of compounds **2a**, **2b**, **2d**, **2e**, **2g**, and **2i** to *h*AChE enzyme active site. The crystal structures of *h*AChE (PDB ID: 4EY7) [22], which was crystallized with donepezil, was retrieved from the Protein Data Bank server (www.pdb.org). The molecular docking procedure was applied as in our previously reported studies [33,34,35,36,37,38,39,40].

## 3. Results and Discussion

### 3.1. Chemistry

In this study, new compounds were synthesized that possessed thiazolylhydrazone moiety. The synthetic route to the obtained compounds **2a**–**2l** is presented in the Scheme 1. Substituted thiosemicarbazone derivatives were obtained by reacting the substituted aldehyde derivatives with thiosemicarbazide. The target compounds (**2a**–**2l**) were synthesized by means of ring closure reaction using thiosemicarbazones (**1a**–**1l**) and appropriate 2-bromoacetophenone.

The final compounds were purified, and their structures were characterized by spectroscopic methods (^1^H NMR, ^13^C NMR, and HRMS). In the ^1^H NMR spectrum, methinic protons had singlet peaks between 7.54 and 7.59 ppm. Thiazole NH exhibited broadened singlet peaks between 12.04 and 12.35 ppm. In the ^13^C NMR spectrum, peak splitting was observed due to the C-F coupling. All of the masses were in accordance with the estimated M+H values.

### 3.2. Anticholinesterase Enzymes Inhibition Assay

All of the attained 3-((2-(4-(4-(trifluoromethyl)phenyl)thiazol-2-yl)hydrazineylidene)methyl)-substituedphenyl derivatives were investigated with regard to their ability to inhibit the activity of cholinesterase enzymes using the in vitro modified Ellman’s spectrophotometric method previously described [33,34,35,36,37,38,39,40].

First, all of the synthesized compounds and reference agents, namely donepezil and tacrine, were subjected to enzyme inhibition assay and prepared at concentrations of 10^−3^ and 10^−4^ M; thus, the first step of the assay was completed. The enzyme activity results of this step are presented in Table 1. Next, the selected compounds that displayed an inhibition profile of more than 50% at a concentration of 10^−4^ M were further tested, together with reference agents, at concentrations of 10^−5^ to 10^−9^ M to perform the second step of enzyme inhibition assay. Therefore, the IC_50_ values of the selected compounds and reference agents were calculated. The percent inhibitions (at concentrations of 10^−3^–10^−9^ M) and IC_50_ values are given in Supplementary Material Table S1.

When all of the results were considered, it was observed that none of the synthesized compounds could pass the second step of the enzyme activity assay in terms of BChE enzyme. Only compounds **2a**, **2b**, **2d**, **2e**, **2g**, **2i**, and **2j** exhibited more than 50% inhibitory activity at a concentration of 10^−3^ M against the BChE enzyme. All of the obtained compounds displayed selective inhibition on the AChE. All of the compounds exhibited more than 50% inhibitory activity at a concentration of 10^−3^ M, whereas compounds **2a**, **2b**, **2d**, **2e**, **2g**, **2i**, and **2j** could pass the second step of AChE enzyme activity assay, and thus, they were subjected to the detailed inhibition assay. The IC_50_ values of compounds **2a**, **2b**, **2d**, **2e**, **2g**, **2i**, and **2j** were calculated as 0.063 ± 0.003, 0.056 ± 0.002, 0.147 ± 0.006, 0.040 ± 0.001, 0.031 ± 0.001, 0.028 ± 0.001, and 0.138 ± 0.005 µM, respectively. It was seen that compounds **2a**, **2b**, **2e**, **2g**, and **2i** had a better AChE enzyme inhibition profile than compounds **2d** and **2j**. Among these derivatives, compound **2i** was found to be the most active agent in the series with an IC_50_ value of 0.028 ± 0.001 µM. It was seen that compound **2i** showed an inhibition profile at a similar rate as the reference drug, donepezil (IC_50_ value = 0.021 ± 0.001 µM).

When the most active compounds **2a**, **2b**, **2e**, **2g**, and **2i** were analyzed chemically, it was seen that these compounds carried the substituents at the 3rd or 4th or 3rd/4th positions of the phenyl ring. Compounds **2d** and **2j**, which showed a moderate inhibition profile, carried the substituents at the 2nd and 2nd/5th positions, respectively. On the other hand, the other inactive compounds **2c**, **2f**, **2h**, **2k**, and **2l** carried the substituents at the 2nd/3rd or 3rd/4th/5th positions of the phenyl ring. It could be suggested that the substituents at the 3rd or 4th or 3rd/4th positions of the phenyl ring activated the compounds biologically. However, the 2nd, 2nd/3rd, 2nd/5th, and 3rd/4th/5th positions resulted in a decrease in enzyme inhibition. Specifically, it was believed that the 2nd position and carrying trisubstituents formed steric obstacles conformationally and thus, this situation results in important interactions belonging to these positions to not be created.

### 3.3. Kinetic Studies of Enzyme Inhibition

**Figure 2:**
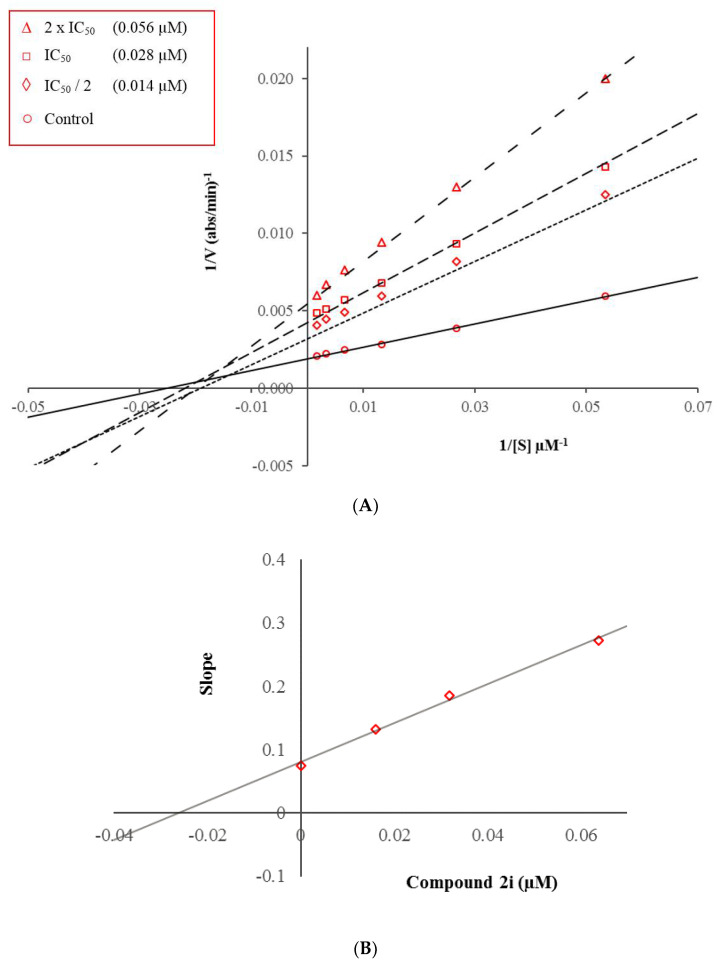
(**A**) Lineweaver–Burk plots for the inhibition of AChE by compound **2i**. [S], substrate concentration (µM); V, reaction velocity (1/V (abs/min)^−1^). Inhibitor concentrations are shown at the left. (**B**) Secondary plot for the calculation of the steady-state inhibition constant (K_i_) of compound **2i**. K_i_ was calculated as 0.025 µM.

Enzyme kinetics studies were conducted to determine the mechanism of inhibition of AChE using a procedure similar to that of the inhibition assay for cholinesterase enzymes. These studies were performed with compound **2i**, which was found to be the most potent agent. Linear Lineweaver–Burk graphs were used to estimate the type of inhibition of this compound. The velocity curves of the substrates were recorded in the absence and presence of compound **2i**. This compound was prepared for enzyme kinetic studies at concentrations of IC_50_/2, IC_50_, and 2xIC_50_. In each case, the initial velocity measurements were obtained at different substrate (ATC) concentrations ranging from 600 to 18.75 µM. To calculate the K_i_ (intercept on the *x*-axis) values of this compound value, the secondary plots of slope (K_m_/V_max_) versus varying concentrations (0, IC_50_/2, IC_50_, and 2xIC_50_) were created. The graphical analyses of steady-state inhibition data for compound **2i** are shown in Figure 2.

According to the Lineweaver–Burk plots, the type of inhibition consists of two general classes: reversible or irreversible. Mixed-type, uncompetitive, competitive, and noncompetitive inhibition types are included in the reversible inhibition [33,34,35,36,37,38,39,40]. As seen in the Lineweaver–Burk plot of compound **2i** (Figure 2), a graph with lines that do not intersect at the *x*-axis or the *y*-axis was formed. This observation indicated that compound **2i** was a reversible and mixed-type inhibitor with similar inhibition features as the substrates. Moreover, it was understood that this finding was compatible with the nature of enzyme inhibition of donepezil. It is known that donepezil has a mixed-type inhibition mechanism [43]. Furthermore, the Ki value of compound **2i** was calculated as 0.025 µM with the help of a secondary plot.

It is known that reversible enzyme inhibition has advantages compared with the irreversible inhibition type, because non-covalent interactions such as hydrophobic interactions, ionic bonds, and hydrogen bonds between the substrate and the enzyme are in question in the reversible inhibition, and these interactions provide the rapid formation and easy breakage of the enzyme-inhibitor complex. Furthermore, reversible inhibitors have a lower risk of side effects than irreversible inhibitors owing to their non-covalent binding ability. Consequently, it can be said that compound **2i**, whose inhibition type was determined to be reversible and mixed type, has a pharmaceutical significance and advantage in that it is an inhibitor candidate of the AChE enzyme.

### 3.4. Prediction of ADME Parameters and BBB Permeability

For a compound candidate to become a drug, it should have appropriate pharmacokinetics, in addition to possessing good pharmacological activity and a low toxicity profile. It is also beneficial if, along with good activity, the molecules to be exposed to advanced bioactivity tests have strong bioavailability. In this way, only compounds with strong potential and acceptable pharmacokinetic properties are chosen for research on drug development [44]. Estimates of the absorption, distribution, metabolism, and excretion (ADME) profiles of drug candidates have been made within the scope of computer-aided drug development studies throughout recent years. The ADME estimates provide early warning of developmental difficulties that may arise in the formulation, process development, and safety that will increase the time/cost of the development of the drug and delay clinical entry [45]. They also allow for the precise design and interpretation of drug discovery experiments [46]. Therefore, in this study, the ADME parameters of the synthesized compounds (**2a**–**2l**) were evaluated using QikProp 4.8 software (Schrödinger Inc., New York, NY, USA) [41].

In addition to the ADME properties, drug-likeness properties were also estimated using QikProp. The drug-likeness of the compounds was assessed according to Lipinski’s rule of 5 and Jorgensen’s rule of 3 [47,48,49,50]. The calculated ADME parameters are presented in Table 2. It can be seen in Table 2 that all of the parameters are within the reference ranges. In keeping with the rules of 3 and 5, the obtained compounds (**2a**–**2l**) were in accordance with the set parameters, as they did not cause more than one violation. Considering the results of the ADME parameter studies, the synthesized compounds were determined to have pharmacokinetic profiles that may be appropriate for clinical use.

Since the molecules are expected to show activity in the central nervous system (CNS) for anti-Alzheimer’s efficiency, when the results were analyzed in this respect, it was seen that the predicted CNS activity values of the compounds were between 0 and 1. The value of 1 on this scale represents positive activity in the CNS. In addition, the log P and log BB values, which are the guides for the compounds to cross the blood–brain barrier, were in the range of 3.641 to 5.273 and (−0.756) to 0.374, respectively, and they were within the recommended limits [51]. Therefore, it can be considered that the synthesized compounds can exceed the blood–brain barrier (BBB), which is very important for CNS-related drugs.

Considering the results of the ADME and BBB permeability studies, the synthesized compounds were determined to possess pharmacokinetic profiles that may be appropriate for clinical use. 

### 3.5. In Vitro BBB Permeability Assay

The capability of BBB penetration is very important for CNS-related drugs. The most active derivative **2i** was submitted to a PAMPA test to detect its BBB permeability (*P_e_*). The results of this assay can be classified as mentioned in Table 3 [42,52,53]:

According to assay results and above classification, it was found that compound **2i** showed good BBB penetration and thus its ability was evaluated as “**CNS+**”.

### 3.6. Molecular Docking

**Figure 3:**
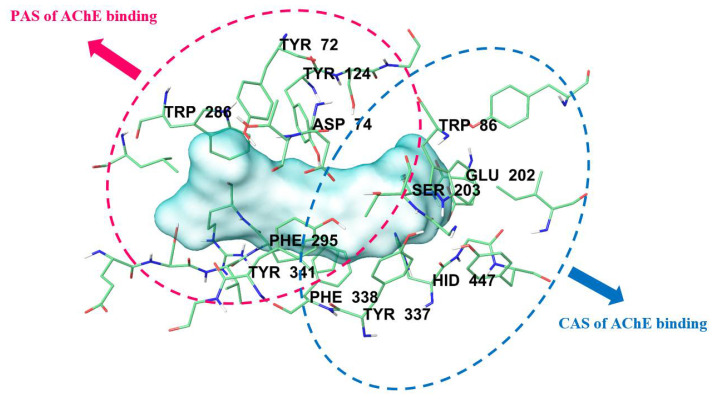
The CAS and PAS regions of AChE enzyme along with the important amino acids.

**Figure 4:**
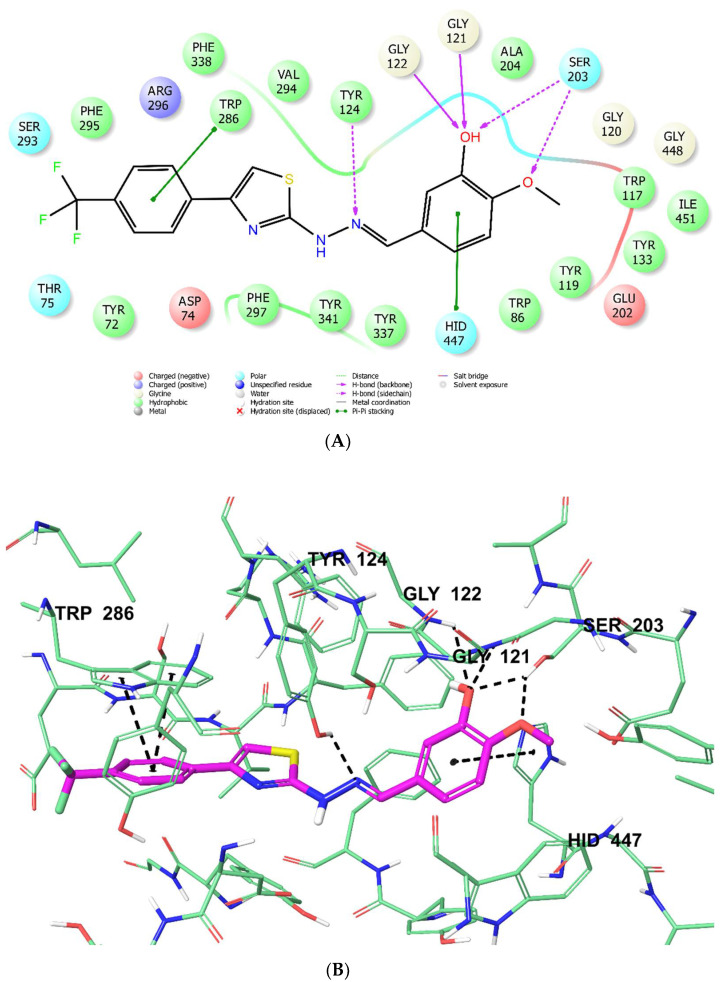
The two- (**A**) and three-dimensional (**B**) interacting mode of compound **2i** in the active region of AChE. The inhibitor and important residues in the active site of enzyme are presented by a tube model colored with purple and aquamarine, respectively (AChE PDB Code: 4EY7).

As described in the inhibition assay for cholinesterase enzymes, compounds **2a**, **2b**, **2e**, **2g**, and **2i** were found to be the most active derivatives against the AChE enzyme in the series. Therefore, docking experiments were conducted to determine their inhibition ability in silico. Based on the docking experiments, further information into the binding modes of compounds **2a**, **2b**, **2e**, **2g**, and **2i** and assessment of the impact of structural modifications on inhibitory activity against the AChE enzyme could be obtained. Studies were carried out using the X-ray crystal structure of *Homo sapiens* AChE (*h*AChE PDB ID:4EY7) [22] retrieved from the Protein Data Bank server (www.pdb.org). This X-ray crystal structure was selected because it is of human nature, has high resolution, and contains a donepezil molecule as the ligand. The rendered docking poses of compounds **2a**, **2b**, **2e**, **2g**, and **2i** are given in Figure 3 and Figure 4, Supplementary Material Figures S1–S5.

According to the crystallographic X-ray structure of the AChE (PDB ID:4EY7), the active pocket enzyme consisted of two major binding sites, the CAS and PAS. The CAS contained the residues of amino acids Trp86, Tyr133, Glu202, Ser203, Tyr337, Phe338, and His447, while amino acids Tyr72, Asp74, Tyr124, Trp286, Phe295, and Tyr341 were identified in the PAS [54,55,56,57] (Figure 3). Donepezil interacts with both the CAS and PAS. Thus, it can be concordantly settled into the gorge due to its dual binding site functionality [58,59]. There is a π–π interaction between the benzyl of donepezil and the indole of Trp86. Moreover, the formation of cation–π interactions are in question between the protonated nitrogen atom of piperidine and the indole of Trp86 and the phenyl of Tyr337. Thus, the benzylpiperidine group of donepezil is strongly located in the CAS. The interactions related to the 1-indanone ring are important for binding to the PAS. In this context, Trp286 amino acid is very essential. The indole of this amino acid creates a π–π interaction with the 1-indanone ring. Furthermore, a hydrogen bond formation is observed between the carbonyl of the 1-indanone and the amine of Phe295 [22,23,24,60,61].

Supplementary Material Figure S1 presents the superimposition of compounds **2a**, **2b**, **2e**, **2g**, and **2i** in the active region of the AChE. The 2- and 3-dimensional docking poses of compounds **2a**, **2b**, **2e**, **2g**, and **2i** are given in Figure 4, Supplementary Material Figures S2–S5. As can be seen from the docking studies, the designed and synthesized compounds in this study can be located in the active region, similar to donepezil. According to these poses, the phenyl of the 4-(4-(trifluoromethyl)phenyl)thiazole group creates a π–π interaction with the indole of Trp286 in compounds **2e**, **2g**, and **2i**. This π–π interaction with Trp286 was observed with the thiazole ring of compound **2a**. For compound **2b**, both of the phenyl and thiazole rings of the 4-(4-(trifluoromethyl)phenyl)thiazole group established π–π interactions with the phenyl of Tyr341. Moreover, in compound **2e**, there was a π–π interaction between the thiazole ring and the phenyl of Tyr341. All of these interactions described provided insight into how compounds **2a**, **2b**, **2e**, **2g**, and **2i** bound to the PAS.

When the interactions were analyzed in terms of binding to the CAS, a π–π interaction formation was seen between the substituted phenyl ring and the indole of Trp86 in compounds **2a** and **2b**. For compounds **2e**, **2g**, and **2i**, this interaction was observed with the imidazole of Hid447. Furthermore, the phenyl ring of compound **2g** formed the other π–π interactions with the phenyl of Phe338. Another important interaction of binding to the CAS was the formation of a hydrogen bond with the hydroxyl of Tyr124. This interaction was seen with the nitrogen of hydrazine moiety in compounds **2a** and **2i**.

The main chemical structural difference of compounds **2a**, **2b**, **2e**, **2g**, and **2i** was the various substituents of the phenyl ring. Compounds **2a** and **2b** had hydroxyl at the 3rd and 4th positions of the phenyl, respectively. The hydroxyl of compound **2a** created a hydrogen bond with the carbonyl of Asp74; however, two hydrogen bond formations were seen between the hydroxyl in compound **2b** and the amino of Gly120 and the carbonyl of Glu202, respectively. Compound **2e** bore a methoxy moiety at the 3rd position of the phenyl, which was different than that of compounds **2a** and **2b**. This methoxy established two hydrogen bonds with the amino of Gly121 and the hydroxyl of Ser203. There were two methoxy moieties at the 3rd and 4th positions of the phenyl in compound **2g**. Each of them formed two hydrogen bonds with the hydroxyl of Ser203. Compound **2i** contained hydroxyl and methoxy moieties at the 3rd and 4th positions of the phenyl, respectively. The hydroxyl of compound **2i** created three hydrogen bonds with the hydroxyl of Ser203 and the amino groups of Gly121 and Gly122. Another hydrogen bond was found between the methoxy at the 4th position of the phenyl and the hydroxyl of Ser203.

Consequently, molecular docking studies have shown that the substituents at the 3rd and 4th positions of the phenyl ring contribute positively to enzyme activity. This result supported the finding that compounds **2g** and **2i**, which had disubstituents at the 3rd and 4th positions, displayed a more potent inhibition profile than compounds **2a**, **2b**, and **2e**, which carried the substituents at the 3rd or 4th positions. Moreover, the reason why compound **2i** was more active than compound **2g** was that it had additional formations of hydrogen bonds belonging to the substituents at the 3rd and 4th positions.

**Figure 5:**
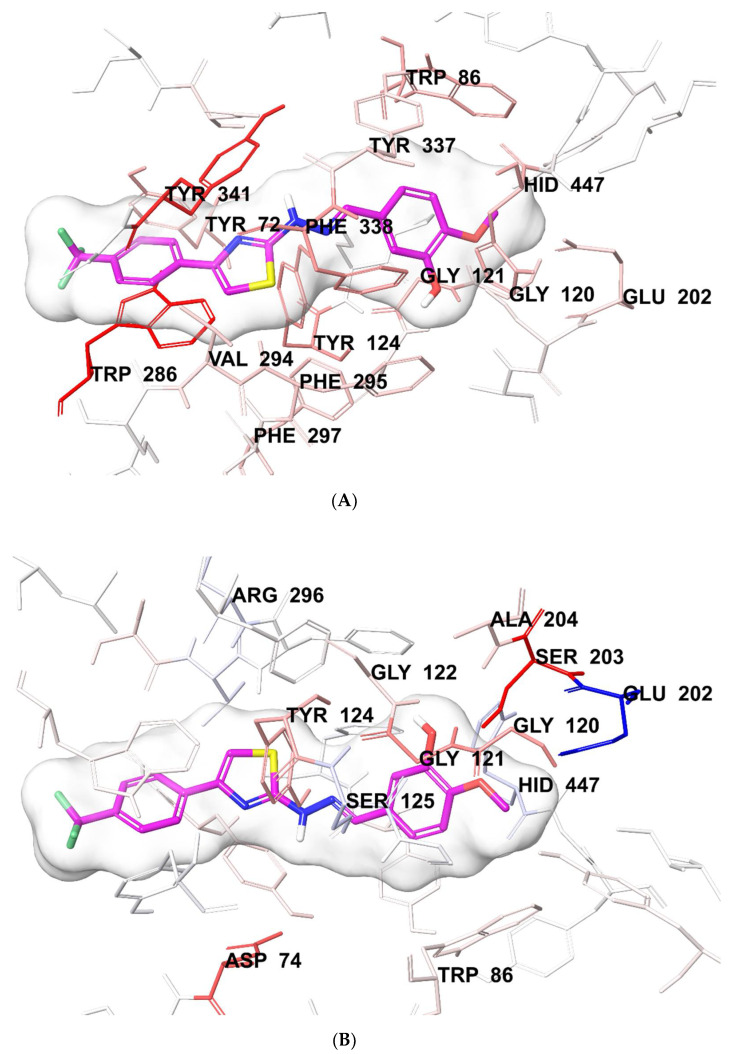
The van der Waals (**A**) and electrostatic interactions (**B**) of compound **2i** with an active region of AChE. The active ligand has a lot of favorable van der Waals and electrostatic interactions. The residues are colored (blue, red, and pink) according to the distance from ligand by per-residue interaction panel.

Molecular docking studies were carried out in detail using the per-residue interaction panel of Glide to determine the contribution of van der Waals and electrostatic interactions in binding to the enzyme active region. Figure 5 presents the van der Waals and electrostatic interactions of compound **2i**, which was found to be the most active agent. It can be seen that this compound had favorable van der Waals interactions with amino acids Tyr72, Trp86, Gly120, Gly121, Tyr124, Glu202, Trp286, Val294, Phe295, Phe297, Tyr337, Phe338, Tyr341, and Hid447, which are displayed in pink and red, as described in the Glide user guide [62]. Similarly, promising electrostatic contributions of compound **2i** were determined with amino acids Asp74, Trp86, Gly120, Gly121, Gly122, Tyr124, Ser125, Glu202, Ser203, Ala204, Arg296, and Hid447. All of these findings explained why compound **2i** exhibited a stronger inhibition profile than the other compounds.

Supplementary Material Figure S6 shows the docking pose of compound **2d**, which displayed a moderate enzyme inhibitory activity in the series. This compound was subjected to molecular docking studies to explain why it was less active than the other compounds (**2a**, **2b**, **2e**, **2g**, and **2i**) and what caused this result chemically. When the docking poses of this compound were analyzed, it was seen that there were common interactions related to the 4-(4-(trifluoromethyl)phenyl)thiazole and benzylidenehydrazine groups in terms of binding to the CAS and PAS regions mentioned above. Compound **2d** had a methoxy moiety at the 2nd position of the phenyl ring, which was different from that of the other compounds (**2a**, **2b**, **2e**, **2g**, and **2i**). However, the hydrogen bond formations observed at the 3rd and 4th positions of other compounds could not be detected at the 2nd position of this compound. The absence of additional interactions belonging to the substituents of the phenyl ring could clarify the decrease in the enzyme inhibitory activity. It was believed that the 2nd position presented a steric obstacle against interaction with any amino acid conformationally. Moreover, it was assumed that this position negatively affected the interaction of the 3rd/4th positions of the compounds, which had disubstituents (at the 2nd and 5th positions) and trisubstituents (at the 3rd, 4th, and 5th positions). Therefore, these compounds could only exhibit interactions related to common chemical structures and could not display remarkable enzyme inhibition profiles such as with compounds **2a**, **2b**, **2e**, **2g**, and **2i**.

## 4. Conclusions

The designed 3-((2-(4-(4-(trifluoromethyl)phenyl)thiazol-2-yl)hydrazineylidene)methyl)-substituedphenyl derivatives as AChE inhibitors were successfully synthesized and analyzed in detail using ^1^H NMR, ^13^C NMR, and HRMS spectrometric techniques. It was also concluded that the synthesized compounds presented promising drug-like characteristics and ADME properties with the help of ADME predictions by using QikProp 4.8 software. Their in vitro enzyme inhibitory activity test results showed that none of the compounds exhibited significant enzyme inhibition against the BChE enzyme. Compounds **2a**, **2b**, **2e**, **2g**, and **2i** containing the substituents at the 3rd or 4th or 3rd/4th positions of the phenyl ring displayed the highest AChE inhibitory activity with the IC_50_ values of 0.063 ± 0.003, 0.056 ± 0.002, 0.040 ± 0.001, 0.031 ± 0.001, and 0.028 ± 0.001 µM, respectively. This is thought to be caused by that the substituents at the 3rd or 4th or 3rd/4th positions of the phenyl ring, which activated the compounds biologically. Specially, compound **2i** displayed a strong inhibitory activity against AChE (IC_50_ = 0.028 ± 0.001 µM), which had an inhibition profile at a similar rate as the reference drug, donepezil (IC_50_ value = 0.021 ± 0.001 µM). This superiority of this compound could be explained by the molecular modeling studies. According to the results of docking studies, compound **2i** bounded to the active site by forming additional interactions via its substituent at the 3rd and 4th positions with Gly121, Gly122, and Ser203 amino acids. In summary, structural modifications can be further made on the basis of the new thiazolylhydrazone derivatives to seek compounds having higher inhibitory activity against ChE enzymes. All these findings encourage medicinal chemists to explore more structures based on thiazolylhydrazone derivatives for high-efficiency ChE inhibitors. New chemical modifications can be designed based on this paper so that novel effective derivatives may be subject to future studies. Hence, studies to develop new candidates that may be effective in Alzheimer’s disease can be followed rationally.

## Figures and Tables

**Table 1:** % Inhibition and IC_50_ values of the synthesized compounds, donepezil and tacrine against AChE and BChE enzymes.

Compounds	AChE % Inhibition		AChE IC_50_ (µM)	BChE % Inhibition		BChE IC_50_ (µM)
	10^−3^ M	10^−4^ M		10^−3^ M	10^−4^ M	
**2a**	93.425 ± 1.652	90.465 ± 1.322	0.063 ± 0.003	52.380 ± 0.935	42.466 ± 0.978	>100
**2b**	90.285 ± 1.451	82.151 ± 1.478	0.056 ± 0.002	55.488 ± 0.875	40.541 ± 0.710	>100
**2c**	78.215 ± 1.025	45.108 ± 0.971	>100	43.612 ± 0.955	32.466 ± 0.721	>1000
**2d**	89.462 ± 2.041	82.445 ± 1.695	0.147 ± 0.006	51.950 ± 0.994	43.795 ± 0.895	>100
**2e**	90.611 ± 1.815	85.387 ± 1.730	0.040 ± 0.001	60.150 ± 1.039	41.575 ± 0.907	>100
**2f**	71.658 ± 1.395	40.487 ± 0.890	>100	40.858 ± 0.895	31.495 ± 0.820	>1000
**2g**	93.461 ± 1.632	88.347 ± 1.604	0.031 ± 0.001	63.515 ± 1.155	40.388 ± 0.845	>100
**2h**	77.561 ± 1.758	41.947 ± 0.976	>100	45.795 ± 0.926	34.306 ± 0.738	>1000
**2i**	95.207 ± 1.502	92.130 ± 1.798	0.028 ± 0.001	65.015 ± 1.470	41.518 ± 0.870	>100
**2j**	91.326 ± 2.107	84.945 ± 1.369	0.138 ± 0.005	50.811 ± 0.988	39.628 ± 0.865	>100
**2k**	65.298 ± 1.045	40.825 ± 0.940	>100	40.171 ± 0.902	25.631 ± 0.633	>1000
**2l**	62.797 ± 1.277	35.369 ± 0.895	>100	38.654 ± 0.815	23.208 ± 0.759	>1000
**Donepezil**	99.254 ± 2.104	97.426 ± 1.890	0.021 ± 0.001	-	-	-
**Tacrine**	-	-	-	98.255 ± 1.895	95.465 ± 1.344	0.006 ± 0.0002

**Table 2:** Calculated ADME (absorption, distribution, metabolism, elimination) parameters of compounds **2a**–**2l**.

Comp.	MW	RB	DM	MV	DHB	AHB	PSA	logP	logS	PCaco	logBB	PMDCK	CNS	PM	%HOA	VRF	VRT
**2a**	363.357	5	6.352	1073.112	2	4.750	57.634	4.295	−6.085	1363.075	−0.292	5986.232	0	2	100	0	1
**2b**	363.357	5	8.087	1073.112	2	4.750	57.634	4.295	−6.085	1363.095	−0.292	5986.324	0	2	100	0	1
**2c**	379.356	6	6.600	1090.479	3	5.500	77.329	3.641	−5.694	602.692	−0.756	2477.781	1	3	100	0	0
**2d**	377.383	5	7.236	1122.809	1	4.750	42.093	5.142	−6.596	4525.117	0.293	10,000	1	2	100	1	1
**2e**	377.383	5	6.313	1113.128	1	4.750	43.433	5.066	−6.219	4525.117	0.313	10,000	1	2	100	1	1
**2f**	407.410	6	5.347	1189.498	1	5.500	50.440	5.192	−6.655	4525.108	0.231	10,000	1	3	100	1	1
**2g**	407.410	6	7.535	1179.309	1	5.500	51.624	5.098	−6.351	4525.108	0.245	10,000	1	3	100	1	1
**2h**	391.367	4	7.047	1084.671	1	5.500	53.698	4.576	−5.904	4526.666	0.374	10,000	1	1	100	0	1
**2i**	393.383	6	7.551	1142.737	2	5.500	65.447	4.388	−6.249	1454.418	−0.334	6420.985	0	3	100	0	1
**2j**	393.383	6	6.825	1132.218	2	5.500	64.300	4.374	−5.890	1674.494	−0.220	7477.311	0	3	100	0	1
**2k**	437.436	7	7.596	1265.955	1	6.250	59.089	5.273	−6.619	4525.108	0.177	10,000	1	4	100	1	1
**2l**	423.409	7	5.877	1207.466	2	6.250	73.261	4.414	−6.177	1428.142	−0.386	6295.69	0	4	100	0	1

**MW:** Molecular weight **RB:** Number of rotatable bonds (recommended value: 0–15) **DM:** Computed dipole moment (recommended value: 1–12.5) **MV:** Total solvent-accessible volume (recommended value: 500–2000) **DHB:** Estimated number of hydrogen bond donors (recommended value: 0–6) **AHB:** Estimated number of hydrogen bond acceptors (recommended value: 2–20) **PSA:** Van der Waals surface area of polar nitrogen and oxygen atoms and carbonyl carbon atoms (recommended value: 7–200) **logP:** Predicted octanol/water partition coefficient (recommended value: −2–6.5) **logS:** Predicted aqueous solubility (recommended value: −6.5–0.5) **PCaco:** Predicted apparent Caco-2 cell permeability (recommended value: <25 poor, >500 great) **logBB:** Predicted brain/blood partition coefficient (recommended value: −3–1.2) **PMDCK:** Predicted apparent MDCK cell permeability (recommended value: <25 poor, >500 great) **CNS:** Predicted central nervous system activity on a −2 (inactive) to +2 (active) scale (recommended value: −2 (inactive), +2 (active)) **PM:** Number of likely metabolic reactions (recommended value: 1–8) **%HOA:** Predicted human oral absorption percent (recommended value: >80% is high, <25% is poor) **VRF:** Number of violations of Lipinski’s rule of five. The rules are: MW < 500, logP < 5, DHB ≤ 5, AHB≤ 10, Positive PSA value. **VRT:** Number of violations of Jorgensen’s rule of three. The three rules are: logS > −5.7, PCaco > 22 nm/s, PM < 7.

**Table 3:** Type of blood–brain barrier (BBB) penetration of compound **2i**.

Classification	Type of BBB Permeation	Compound	Type of BBB Permeation
**CNS+**	High BBB permeation *P_e_* (10^−6^ cm s^−1^) > 4.0	**2i**	**CNS+** High BBB permeation
**CNS>−**	Low BBB permeation *P_e_* (10^−6^ cm s^−1^) < 2.0		
**CNS±**	BBB permeation uncertain 2.0 < *P_e_* (10^−6^ cm s^−1^) < 4.0		

## Supplementary Materials

The following are available online, **Table S1.** IC_50_ values of compounds **2a**, **2b**, **2d**, **2e**, **2g**, **2i**, **2j** and donepezil against AChE. **Figure S1.** The superimposition pose of selected compounds in the enzyme active site (AChE PDB Code: 4EY7). **Figure S2.** The two- (**A**) and three-dimensional (**B**) interacting mode of compound **2a** in the active region of AChE. The inhibitor and important residues in the active site of enzyme are presented by tube model and colored with grey and aquamarine, respectively (AChE PDB Code: 4EY7). **Figure S3.** The two- (**A**) and three-dimensional (**B**) interacting mode of compound **2b** in the active region of AChE. The inhibitor and important residues in the active site of enzyme are presented by tube model and colored with blue and aquamarine, respectively (AChE PDB Code: 4EY7). **Figure S4.** The two- (**A**) and three-dimensional (**B**) interacting mode of compound **2e** in the active region of AChE. The inhibitor and important residues in the active site of enzyme are presented by tube model and colored with orange and aquamarine, respectively (AChE PDB Code: 4EY7). **Figure S5.** The two- (**A**) and three-dimensional (**B**) interacting mode of compound **2g** in the active region of AChE. The inhibitor and important residues in the active site of enzyme are presented by tube model and colored with dark green and aquamarine, respectively (AChE PDB Code: 4EY7). **Figure S6.** The two- (**A**) and three-dimensional (**B**) interacting mode of compound **2d** in the active region of AChE. The inhibitor and important residues in the active site of enzyme are presented by tube model and colored with pink and aquamarine, respectively (AChE PDB Code: 4EY7). **Figure S7.** The thin-layer chromatography of the synthesized compounds. **Figure S8.** Purity of compound **2i**. **Figure S9.** ^1^H NMR spectra of the compound **2i**. **Figure S10.** ^13^C NMR spectra of the compound **2i**. **Figure S11.** HRMS spectra of the compound **2i**.
