# Supplementary material for: Design, Synthesis, and Structure–Activity Relationships of Thiazole Analogs as Anticholinesterase Agents for Alzheimer’s Disease
Source: Molecules. 2020 Sep 20;25(18):4312. doi: 10.3390/molecules25184312 (PMC7570694; doi:10.3390/molecules25184312)
Supplement: Supplementary file 1 [file molecules-25-04312-s001.pdf]

SUPPORTING INFORMATION

Design, Synthesis, and Structure–Activity Relationships of Thiazole Analogs as Anticholinesterase Agents for Alzheimer's Disease

Begüm Nurpelin Sağlık [1,2], Derya Osmaniye [1,2], Ulviye Acar Çevik [1,2], Serkan Levent [1,2], Betül Kaya Çavuşoğlu [3], Yusuf Özkay [1,2] and Zafer Asım Kaplancıklı [1*]

[1] Department of Pharmaceutical Chemistry, Faculty of Pharmacy, Anadolu University, 26470 Eskişehir, Turkey
[2] Doping and Narcotic Compounds Analysis Laboratory, Faculty of Pharmacy, Anadolu University, 26470 Eskişehir, Turkey
[3] Department of Pharmaceutical Chemistry, Faculty of Pharmacy, Zonguldak Bülent Ecevit University, 67600 Zonguldak, Turkey
* Correspondence: zakaplan@anadolu.edu.tr; Tel.: +90-222-335-0580/3777

Table S1. IC_{50} values of compounds **2a**, **2b**, **2d**, **2e**, **2g**, **2i**, **2j** and donepezil against AChE.

**Compounds**	**AChE % Inhibition**							**IC_{50}**
	**10^{-3} M**	**10^{-4} M**	**10^{-5} M**	**10^{-6} M**	**10^{-7} M**	**10^{-8} M**	**10^{-9} M**	**(μM)**
**2a**	93.425 ±1.652	90.465 ±1.322	81.239 ±1.421	74.155 ±1.518	60.975 ±1.182	41.275 ±0.954	20.388 ±0.711	0.063 ±0.003
**2b**	90.285 ±1.451	82.151 ±1.478	74.565 ±1.279	63.758 ±1.230	57.965 ±1.023	43.948 ±0.963	21.208 ±0.839	0.056 ±0.002
**2d**	89.462 ±2.041	82.445 ±1.695	78.103 ±1.766	67.321 ±1.159	55.922 ±1.004	38.162 ±0.847	29.751 ±0.855	0.147 ±0.006
**2e**	90.611 ±1.815	85.387 ±1.730	81.828 ±1.311	74.797 ±1.488	62.356 ±1.103	44.275 ±0.904	21.599 ±0.631	0.040 ±0.001
**2g**	93.461 ±1.632	88.347 ±1.604	81.465 ±1.752	76.911 ±1.326	69.188 ±1.055	43.104 ±0.952	24.903 ±0.789	0.031 ±0.001
**2i**	95.207 ±1.502	92.130 ±1.798	86.602 ±1.214	80.964 ±1.124	71.840 ±1.270	42.651 ±0.866	22.155 ±0.830	0.028 ±0.001
**2j**	91.326 ±2.107	84.945 ±1.369	76.841 ±1.830	62.108 ±1.411	57.365 ±1.036	40.715 ±0.949	25.575 ±0.899	0.138 ±0.005
**Donepezil**	99.254 ±2.104	97.426 ±1.890	92.258 ±1.510	90.318 ±1.104	81.365 ±1.104	43.875 ±0.601	21.418 ±0.548	0.021 ±0.001

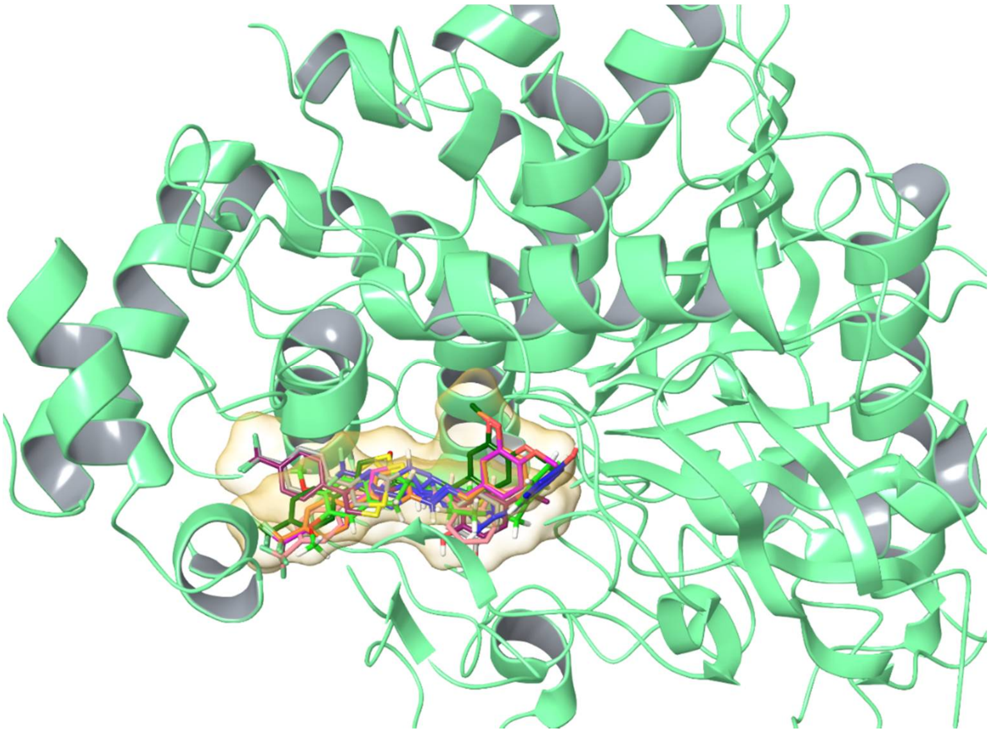

Figure S1. The superimposition pose of selected compounds in the enzyme active site (AChE PDB Code: 4EY7).

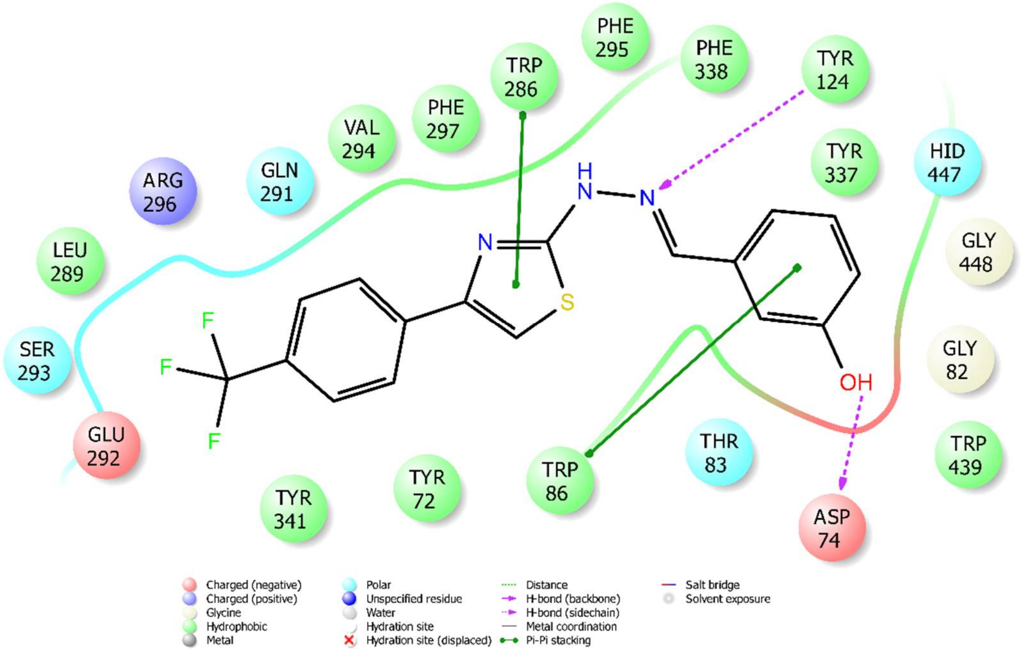

A

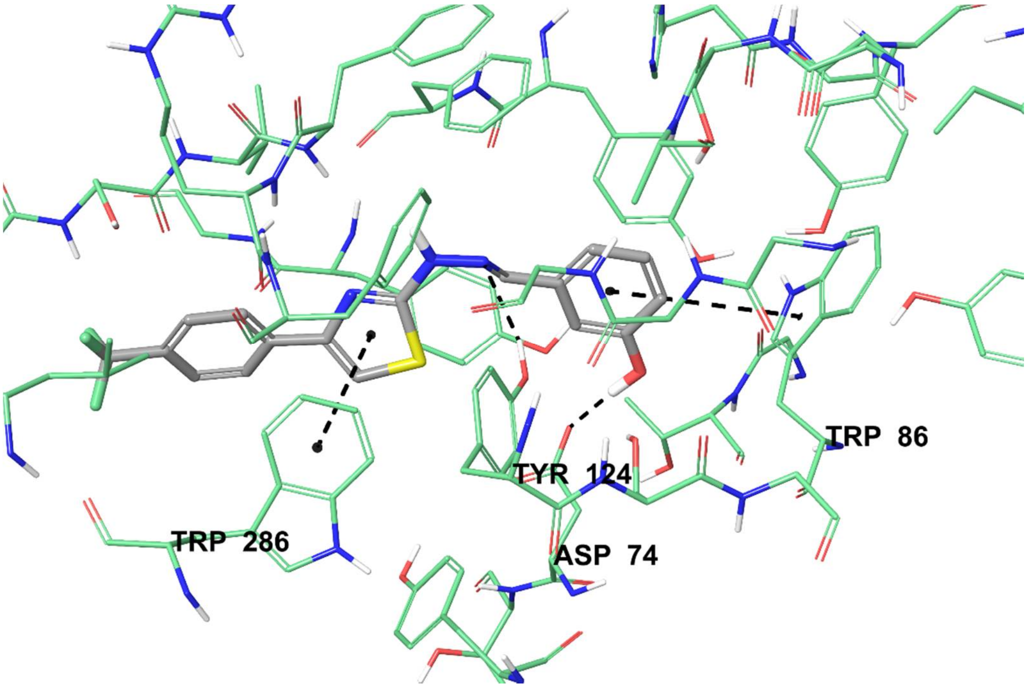

B

Figure S2. The two- (**A**) and three-dimensional (**B**) interacting mode of compound **2a** in the active region of AChE. The inhibitor and important residues in the active site of enzyme are presented by tube model and colored with grey and aquamarine, respectively (AChE PDB Code: 4EY7).

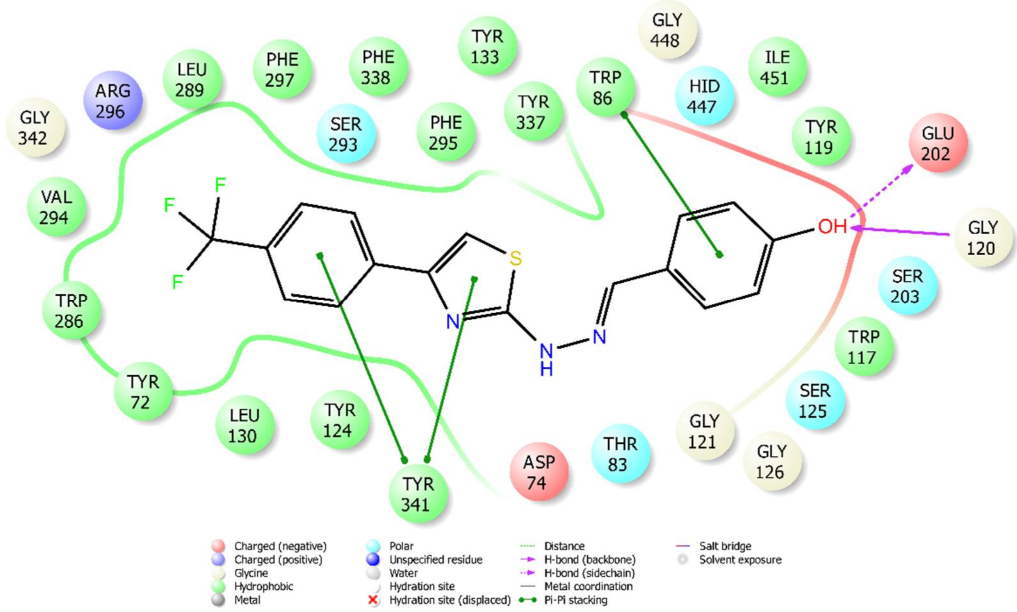

A

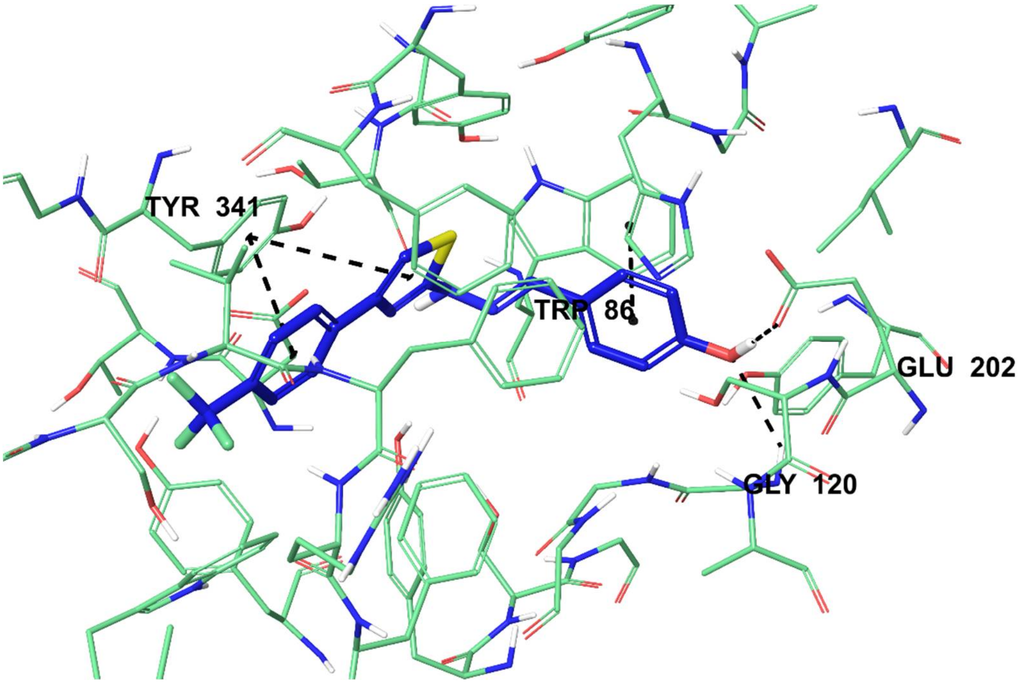

B

Figure S3. The two- (**A**) and three-dimensional (**B**) interacting mode of compound **2b** in the active region of AChE. The inhibitor and important residues in the active site of enzyme are presented by tube model and colored with blue and aquamarine, respectively (AChE PDB Code: 4EY7).

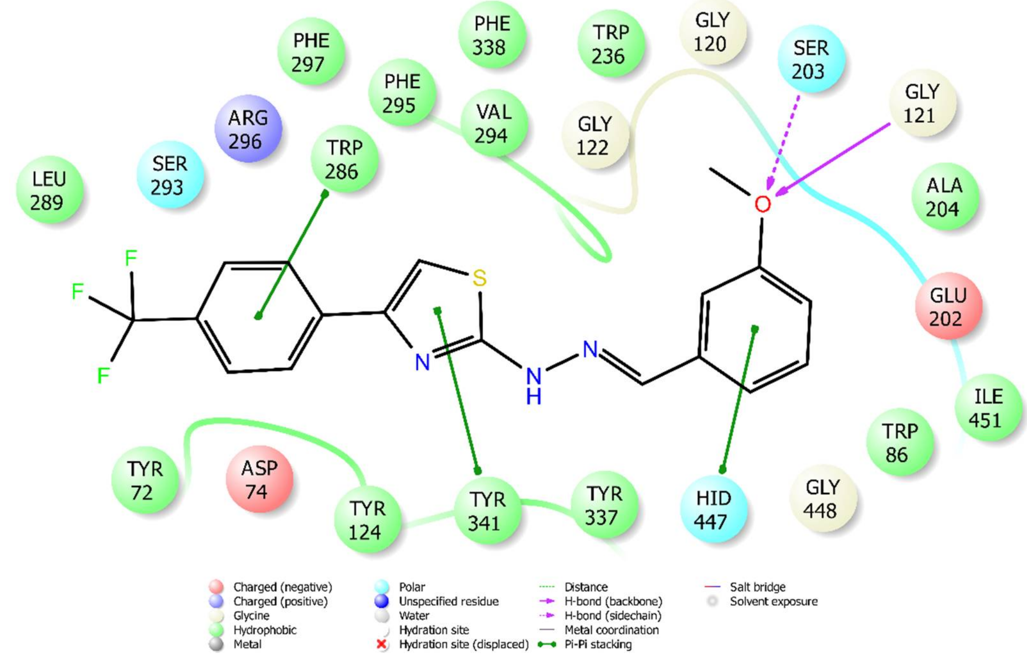

A

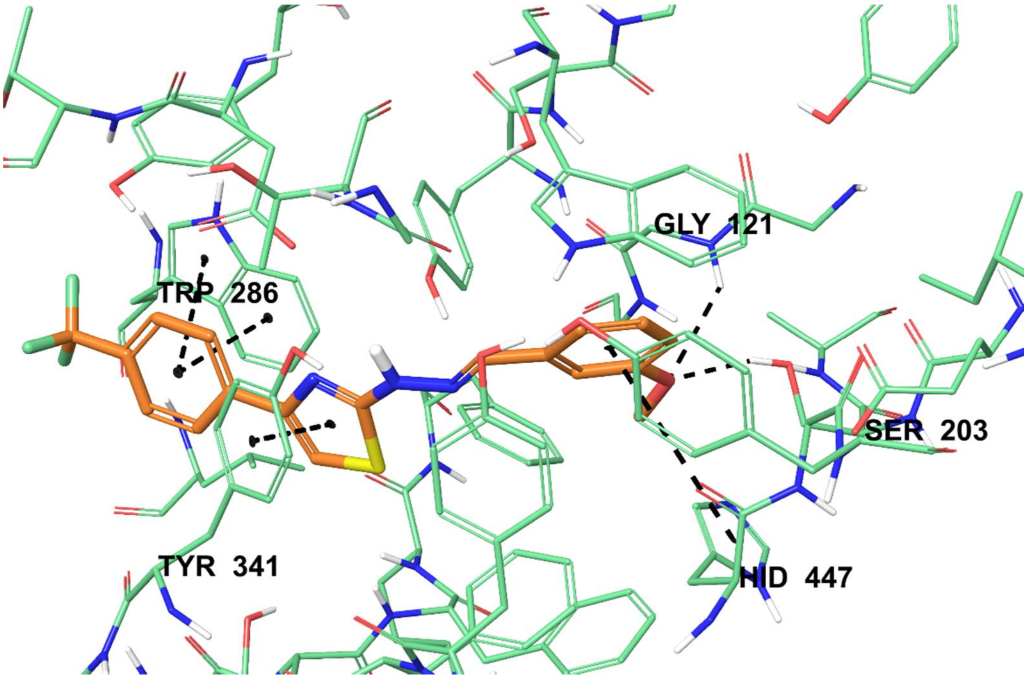

B

Figure S4. The two- (**A**) and three-dimensional (**B**) interacting mode of compound **2e** in the active region of AChE. The inhibitor and important residues in the active site of enzyme are presented by tube model and colored with orange and aquamarine, respectively (AChE PDB Code: 4EY7).

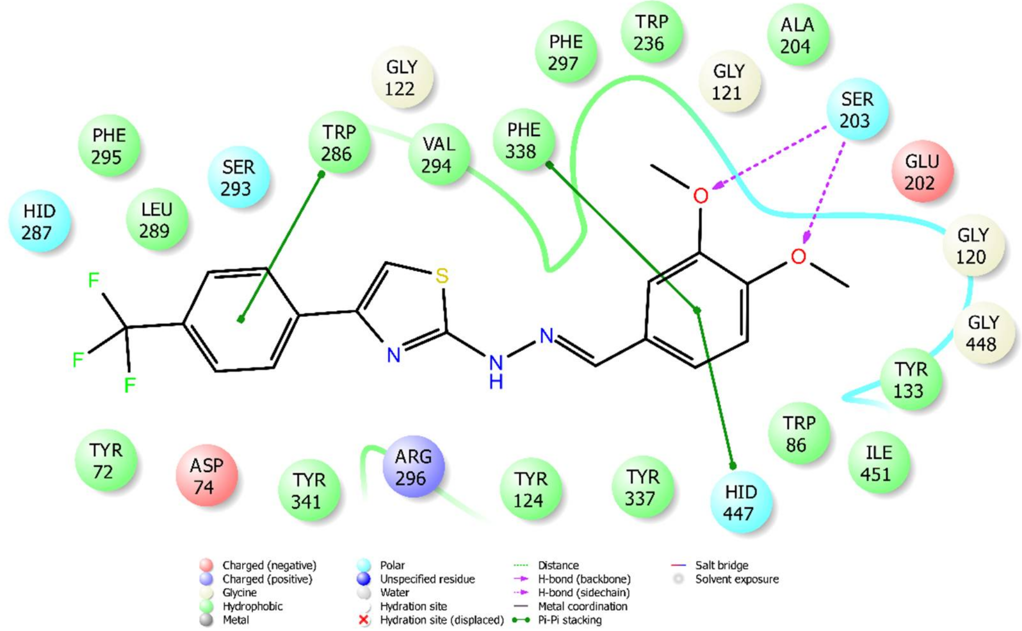

A

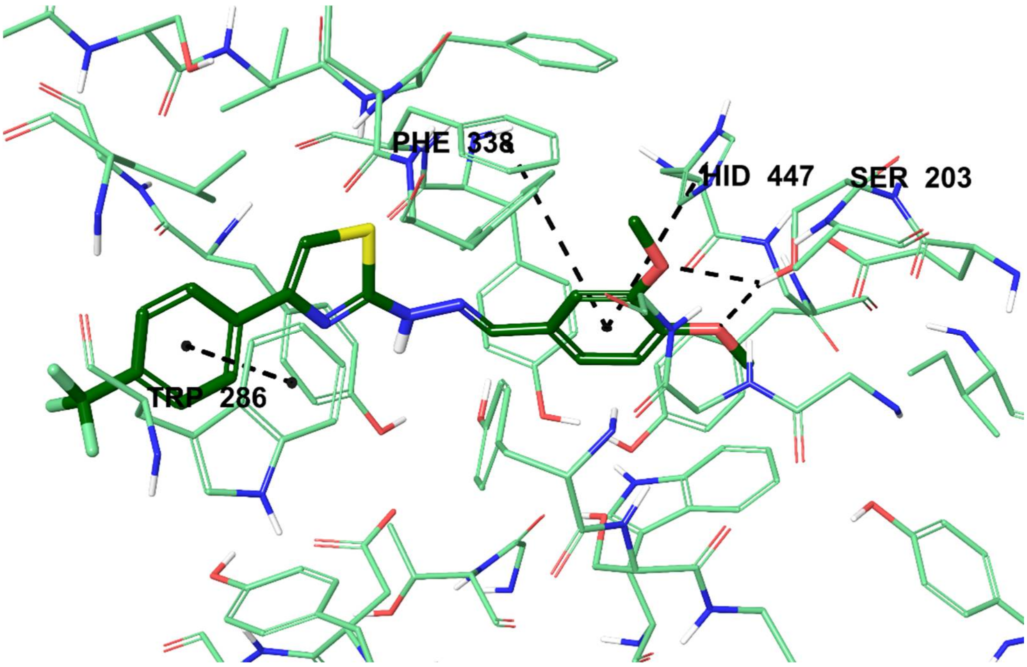

B

Figure S5. The two- (**A**) and three-dimensional (**B**) interacting mode of compound **2g** in the active region of AChE. The inhibitor and important residues in the active site of enzyme are presented by tube model and colored with dark green and aquamarine, respectively (AChE PDB Code: 4EY7).

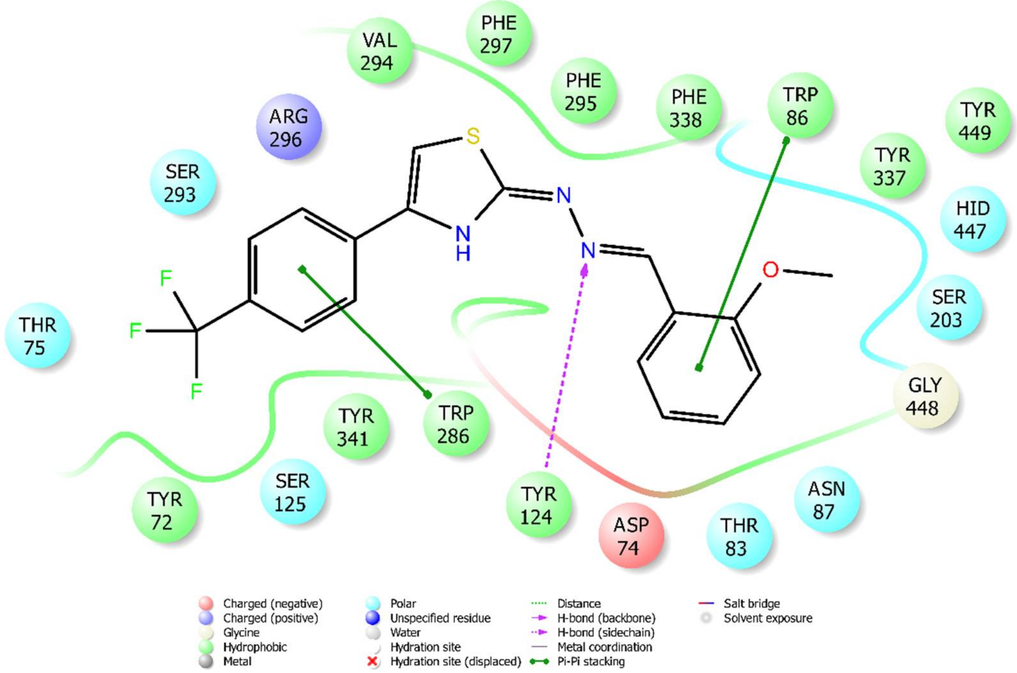

A

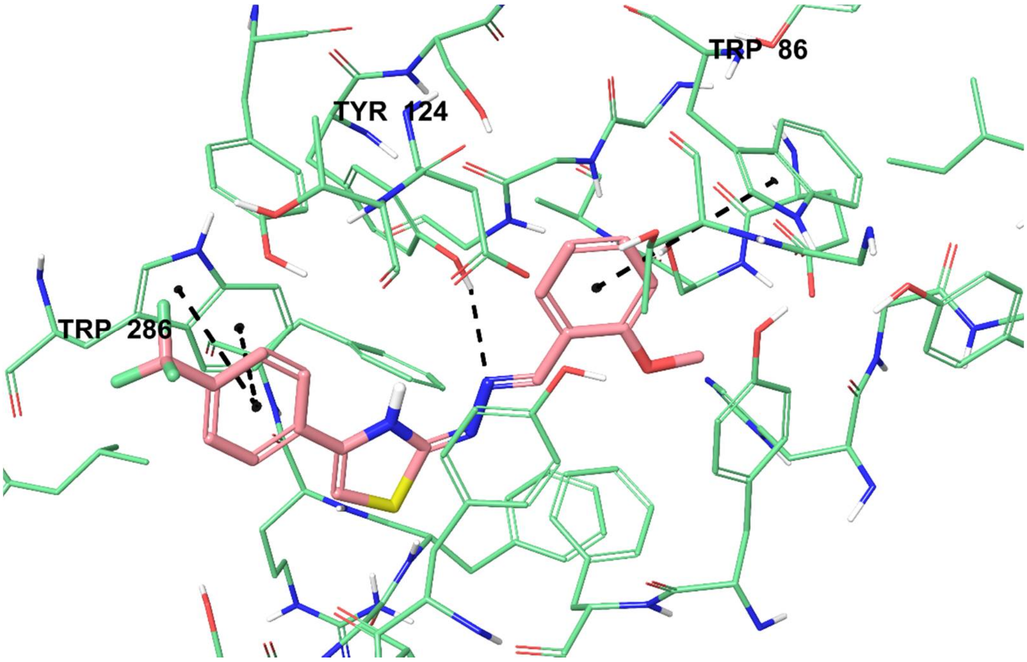

B

Figure S6. The two- (**A**) and three-dimensional (**B**) interacting mode of compound **2d** in the active region of AChE. The inhibitor and important residues in the active site of enzyme are presented by tube model and colored with pink and aquamarine, respectively (AChE PDB Code: 4EY7).

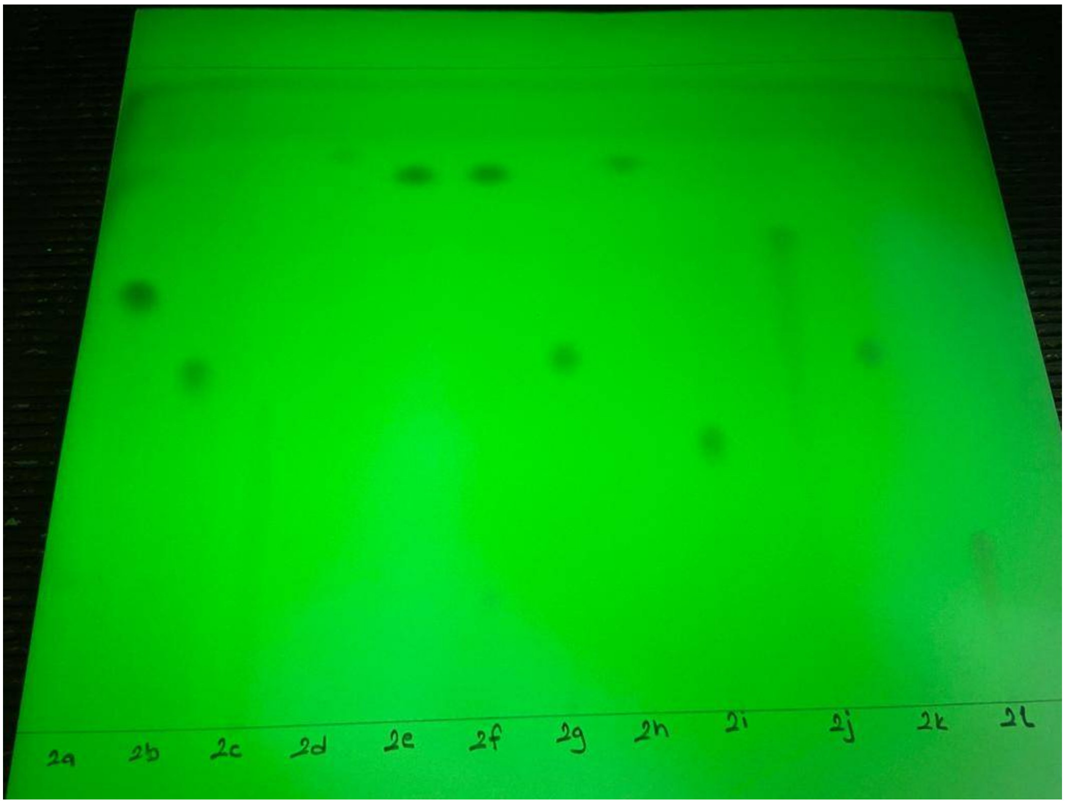

Figure S7. The thin-layer chromatography of the synthesized compounds.

==== Shimadzu LCMSsolution Analysis Report ====

Acquired by : Admin
Sample Name : tf-14
Sample ID :
Vail # : 32
Injection Volume : 0.3 uL
Data File Name : tf-14_43.lcd
Method File Name : isocratic.lcm
Batch File Name : batch.lcb
Report File Name : DefaultLCMS.lcr
Data Acquired : 12.03.2020 01:13:26
Data Processed : 12.03.2020 01:23:28

<Chromatogram>

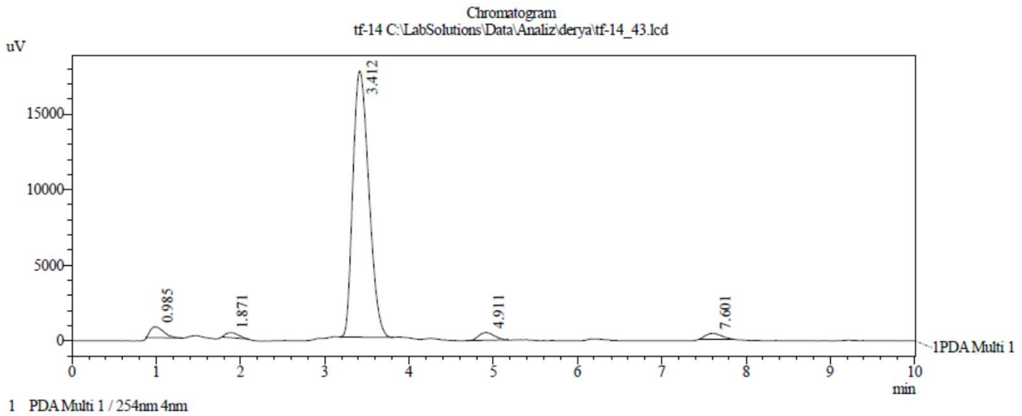

1 PDA Multi 1 / 254nm 4nm

PeakTable

PDA Ch1 254nm 4nm

Peak#	Ret. Time	Area	Height	Area %	Height %
1	0.985	7787	705	3.059	3.608
2	1.871	3512	321	1.380	1.643
3	3.412	232017	17619	91.149	90.187
4	4.911	6455	509	2.536	2.603
5	7.601	4776	383	1.876	1.959
Total		254546	19537	100.000	100.000

Figure S8. Purity of compound **2i**.

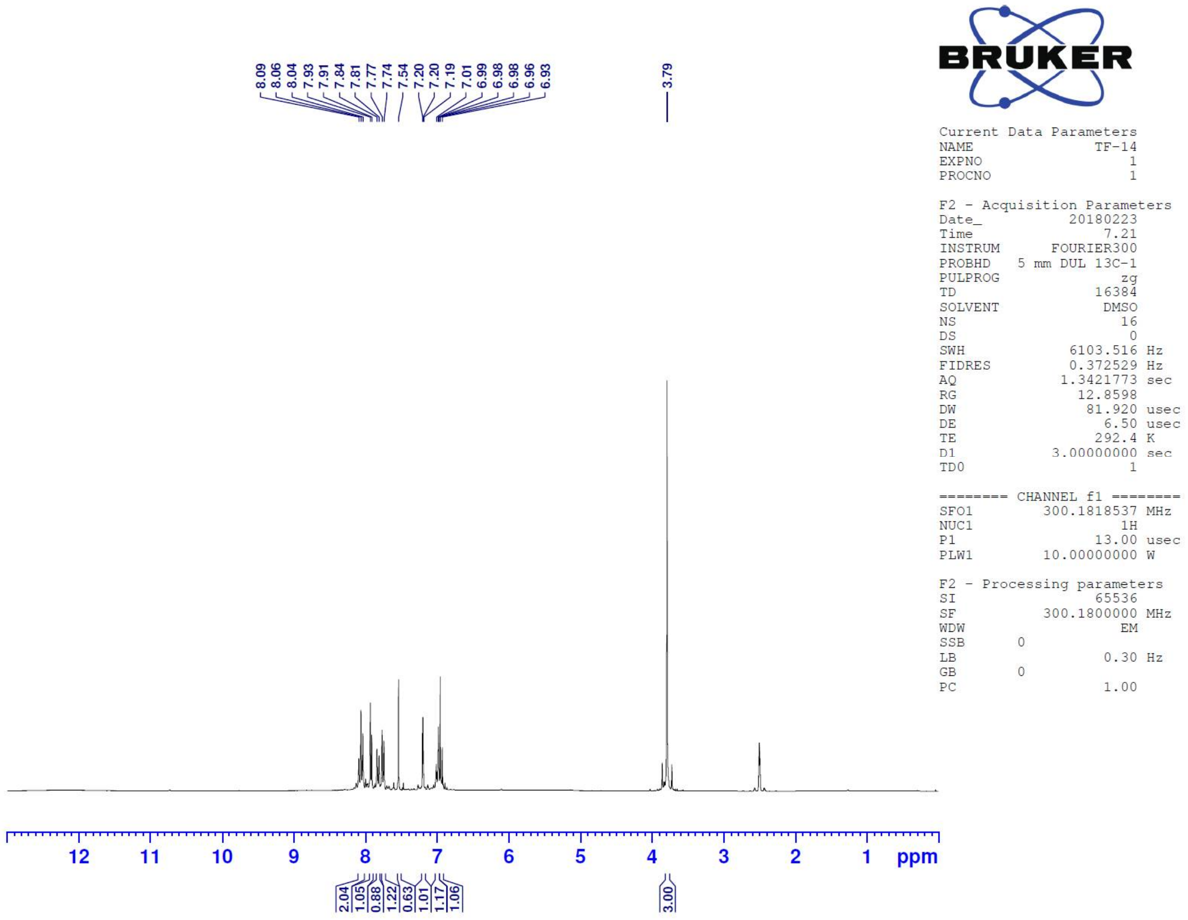

Figure S9. ^{1}H NMR spectra of the compound **2i**.

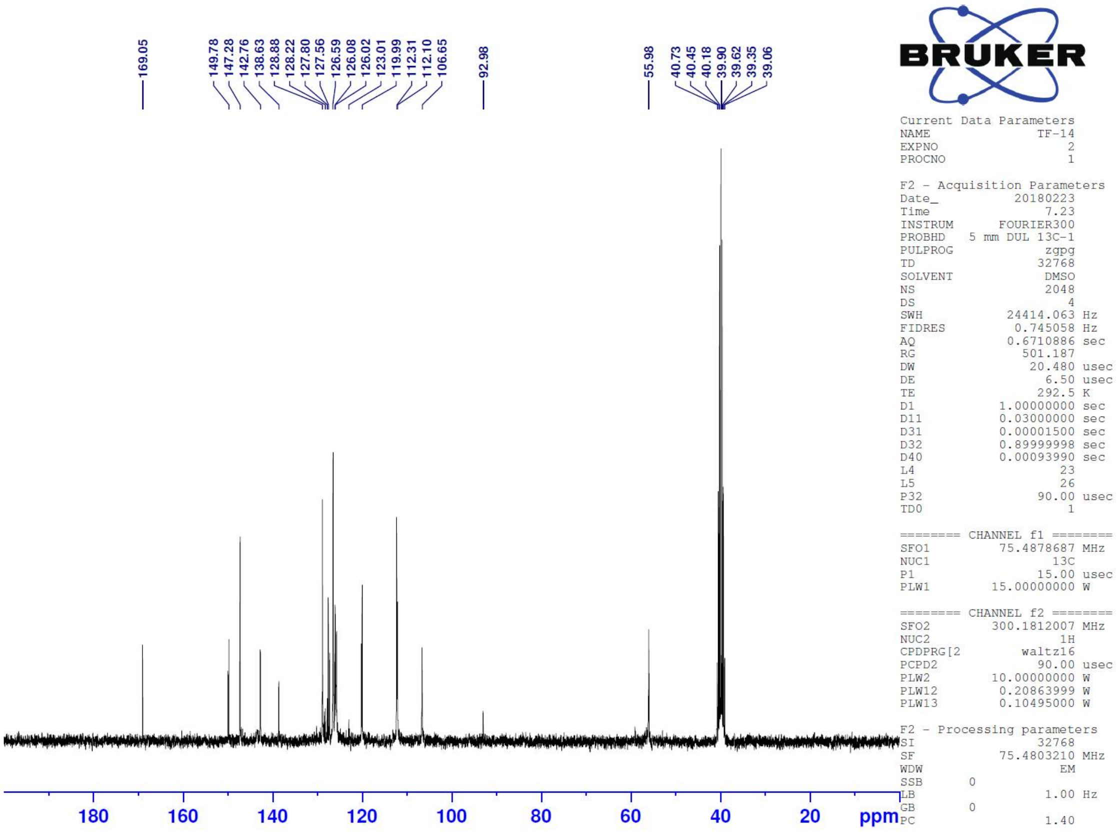

Figure S10. ^{13}C NMR spectra of the compound **2i**.

Data File: C:\LabSolutions\Data\Analiz\derya\tf-14_43.lcd

Elmt	Val.	Min	Max	Elmt	Val.	Min	Max	Elmt	Val.	Min	Max	Elmt	Val.	Min	Max	Use Adduct
H	1	6	40	O	2	1	4	S	2	1	1	Ru	2	0	0	H
C	4	7	33	F	1	3	3	Cl	1	0	0	Pd	2	0	0	
N	3	2	8	P	3	0	0	Br	1	0	0	I	3	0	0	

Error Margin (ppm):	5	DBE Range:	5.0 - 20.0	Electron Ions:	both
HC Ratio:	unlimited	Apply N Rule:	yes	Use MSn Info:	yes
Max Isotopes:	3	Isotope RI (%):	1.00	Isotope Res:	9000
MSn Iso RI (%):	10.00	MSn Logic Mode:	AND	Max Results:	100

Event#: 1 MS(E+) Ret. Time : 3.587 Scan# : 539

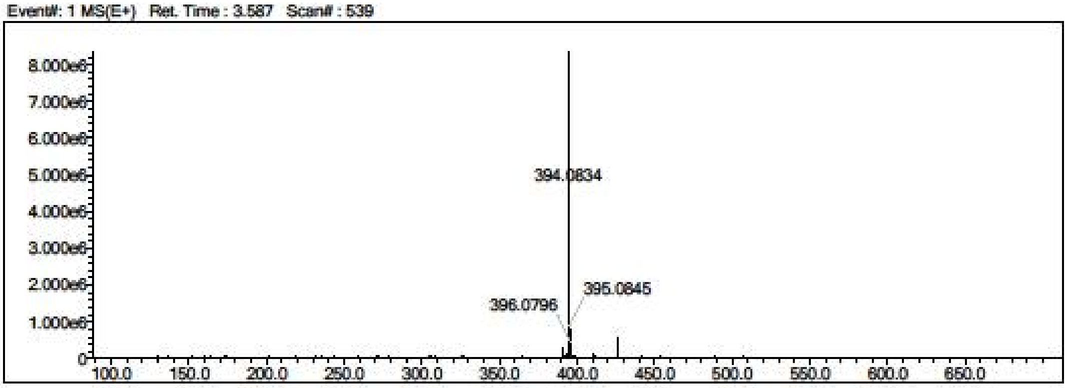

Measured region for 394.0834 m/z

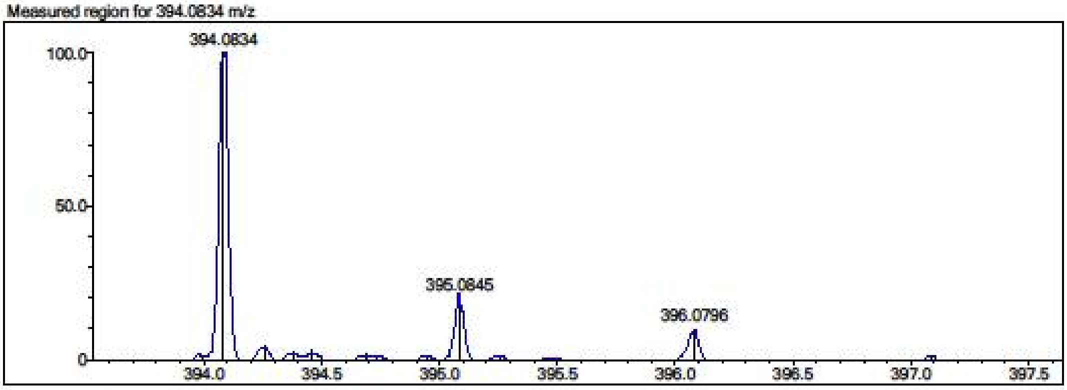

C18 H14 N3 O2 F3 S [M+H]+ : Predicted region for 394.0832 m/z

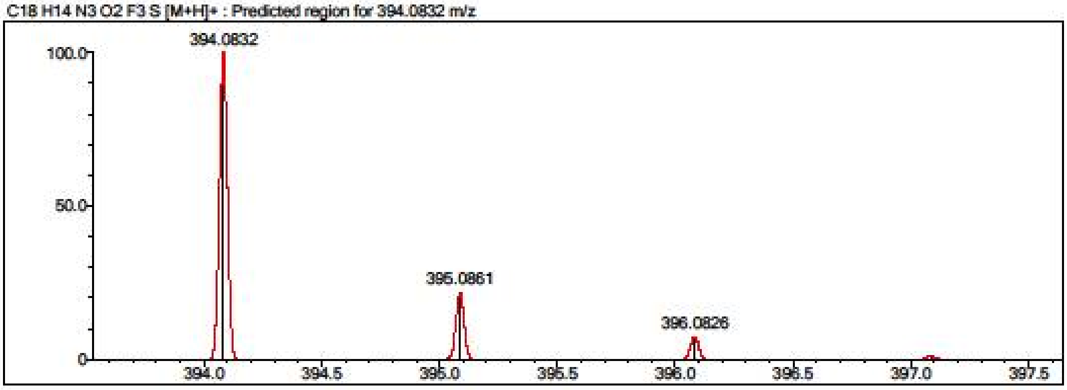

Rank	Score	Formula (M)	Ion	Meas. m/z	Pred. m/z	Df. (mDa)	Df. (ppm)	Iso	DBE
1	95.26	C18 H14 N3 O2 F3 S	[M+H]+	394.0834	394.0832	0.2	0.51	95.26	12.0

Figure S11. HRMS spectra of the compound **2i**.
